# Memory in Microbes: Quantifying History-Dependent Behavior in a Bacterium

**DOI:** 10.1371/journal.pone.0001700

**Published:** 2008-02-27

**Authors:** Denise M. Wolf, Lisa Fontaine-Bodin, Ilka Bischofs, Gavin Price, Jay Keasling, Adam P. Arkin

**Affiliations:** 1 Physical Biosciences Division, Lawrence Berkeley National Laboratory, Berkeley, California, United States of America; 2 Department of Bioengineering, University of California, Berkeley, California, United States of America; 3 Department of Chemical Engineering, University of California, Berkeley, California, United States of America; Harvard Medical School, United States of America

## Abstract

Memory is usually associated with higher organisms rather than bacteria. However, evidence is mounting that many regulatory networks within bacteria are capable of complex dynamics and multi-stable behaviors that have been linked to memory in other systems. Moreover, it is recognized that bacteria that have experienced different environmental histories may respond differently to current conditions. These “memory” effects may be more than incidental to the regulatory mechanisms controlling acclimation or to the status of the metabolic stores. Rather, they may be regulated by the cell and confer fitness to the organism in the evolutionary game it participates in. Here, we propose that history-dependent behavior is a potentially important manifestation of memory, worth classifying and quantifying. To this end, we develop an information-theory based conceptual framework for measuring both the persistence of memory in microbes and the amount of information about the past encoded in history-dependent dynamics. This method produces a phenomenological measure of cellular memory without regard to the specific cellular mechanisms encoding it. We then apply this framework to a strain of *Bacillus subtilis* engineered to report on commitment to sporulation and degradative enzyme (AprE) synthesis and estimate the capacity of these systems and growth dynamics to ‘remember’ 10 distinct cell histories prior to application of a common stressor. The analysis suggests that *B. subtilis* remembers, both in short and long term, aspects of its cell history, and that this memory is distributed differently among the observables. While this study does not examine the mechanistic bases for memory, it presents a framework for quantifying memory in cellular behaviors and is thus a starting point for studying new questions about cellular regulation and evolutionary strategy.

## Introduction

Your average bacterium is unlikely to recite π to 15 places or compose a symphony. Yet evidence is mounting that these ‘simple’ cells contain complex control circuitry capable of generating multi-stable behaviors and other complex dynamics that have been conceptually linked to memory in other systems. And though few would call this phenomenon memory in the ‘human’ sense, it has long been known that bacterial cells that have experienced different environmental histories may respond differently to current conditions [1]–[3]. Though some of these history-dependent behavioral differences may be physically necessary consequences of the prior history, and thus some might argue insignificant, other behavioral differences may be controllable and therefore selectable and even fitness enhancing manifestations of memory.

In this paper we take the potentially controversial view that history-dependent behavior, whether short or long term, controlled or incidental, reflects a form of memory [4]–[6]. Because bacterial dynamics at every level of resolution operate within the limitations and potentials of nonlinear physical and biochemical dynamical systems, they must exhibit at least very short-term transient memory, and potentially longer term memory. The type of memory (and its significance) depends on which features of cell history are ‘remembered’, and at what resolution; whether or not the system eventually ‘forgets’ its past, and if so, how long this forgetting takes; the mechanisms in the cell responsible for memory storage, encoding, and retrieval; and whether or not this memory provides a fitness advantage in a natural environment. In cellular systems, environmental memory has been noted to be inherent in everything from the selective history of mutation, epigenetic inheritance via chromatin modification in neurons and DNA methylation in chemotaxing bacteria [7], genetic and epigenetic phase variation mechanisms controlling surface features of pathogenic bacteria [8], [9], cellular proliferation and survival in the immune system, and in switch-like feedback systems in regulatory networks spanning signal transduction, metabolism and gene expression [10]–[21]. There is also a growing body of work focusing on synthetically designing and constructing network motifs and systems that are capable of showing some types of dynamic memory [22], [23]. These and many other studies in synthetic and natural systems suggest that even the simplest first-order chemical reactions have at least transient memory of initial conditions, and more complex mechanisms involving history-dependent changes in the concentrations, states and localization of proteins and other regulatory network elements can encode a wide range of input information and store it for amounts of time ranging from minutes to days or longer [4], [16], [24], [25]. The state dynamics of such systems contain the memory of past controlling inputs, and even of past environmental conditions if one is to interpret more broadly [5], [26].

In metazoans, the ability of somatic cells to remember their fates is key to development and thus to organismal fitness. The same can be said for other types of metazoan cells like those found in the immune system that use a memory of past states to modify future behavior. In principle at least, memory, whether short- or long-term, can feasibly confer an evolutionary advantage in microbes as well. For instance, Hoffer *et. al.* suggest that in *E. coli* a form of ‘memory’ of past phosphate limitation leads to a faster response to successive periods of phosphate limitation, and that this faster response may be survival enhancing [5]. It has also been suggested that pathogenic bacteria use cross-talk encoded memory to balance the demands of immune avoidance with a sequential, compartment to compartment infection lifecycle [8], [9]. More abstractly, the dynamic implementation of cellular behaviors can be viewed as a selected, ‘winning’ (or at least stable) strategy in an evolutionary game [12], [27]. In game theory, information creates advantage [28]–[30], and information about the past as well as the present creates even greater advantage. Thus if bacterial cells are able to store information about past experience in some type of memory, and use this memory to modulate their behavior, this opens up the possibility of playing game strategies with memory, a provably superior family of strategies compared to those without memory [31]–[35]. Even if the memory capacity of the system is short term, but on the order of environmental fluctuations, it could conceivably impact fitness and therefore play a role in an evolved adaptive behaviour [28].

**Figure 1:**
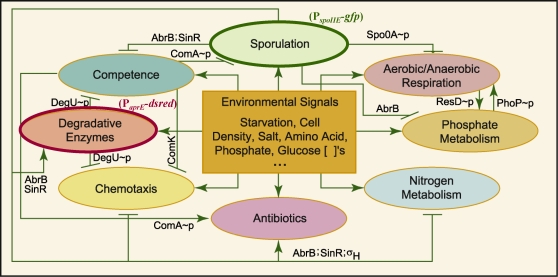
The *B. subtilis* stress response meta-network, where each oval represents both a stress response and the regulatory network of 100 or so interacting molecular species that regulates it. Among the many ingenious genetic and biochemical programs employed by *B. subtilis* to cope with environmentally adverse conditions are its ability to take up extracellular DNA, competence [40], [76]; differentiate into an inert heat-, chemical, and UV-resistant spore [37]; secrete degradative enzymes to identify and digest new food sources [77]; become motile and chemotax toward possibly better surroundings [78]; synthesize antibiotics to eliminate competitors in the same ecosystem [79], [80]; turn on alternative metabolic pathways, and form biofilms ([81], not shown), just to name a few [38]. The cross-repressive feedback between sporulation and competence, and the many positive feedback loops within each large ‘individual’ stress response pathway [10], [36]–[40], [82], are suggestive of switches and other elements that could potentially encode memory. The two stress response pathways monitored in our experiments, sporulation and synthesis of the degradative enzyme subtilisin, are denoted by bold-faced ovals. The fluorescent reporters (GFP and DsRed) fused to the respective promoters P*spoIIE* and P*aprE* are indicated (see Materials and Methods).

Given the potential ubiquity and significance of bacterial memory, we propose that quantifying history dependent behavior in microbes could be an important piece of the puzzle of bacterial regulation, survival strategy, and evolution. To this end, we developed an information-theory based conceptual framework for thinking about and measuring both the persistence of memory in microbes and the amount of information about the past encoded in these dynamics. This method produces a phenomenological measure of cellular memory *without* regard to the specific cellular mechanisms encoding it. We then applied this framework to the bacterium *B. subtilis*. *B. subtilis* presents an excellent model organism for this study because of its exquisite sensitivity to environmental conditions, its known mechanisms of bistability and other hysteretic switch-like regulatory stress response mechanisms and architectures, and its developmental decision to sporulate that strongly resembles eukaryotic memory-associated processes determining developmental cell fate ([10], [36]–[40], Fig. 1). Also, certain aspects of *B. subtilis* behavior, such as spore coat composition, have already been associated with environmental memory [41]–[43], and though much suggests that there should be memory, how these response dynamics depend on past conditions prior to application of a stress has not been systematically examined.

In our experiments, we quantified the ability of three *B. subtilis* stress response systems–sporulation, degradative enzyme synthesis, and growth-to ‘remember’ 10 distinct cell histories prior to application of a common stressor. We chose to observe commitment to sporulation (via reporter fusion to P_SPOIIE_) because the sporulation decision is bistable, and bistability is associated with memory [9], [11], [16], [44]. We added the reporter for degradative enzyme synthesis (measured by a fluorescent reporter fused to the AprE promoter) because though it shares many common controllers with sporulation, its expression pattern is quite different and not believed to be bistable or probabilistic. We wondered whether any history-dependence in sporulation control would be mirrored in AprE control. Finally, we chose to observe growth (as measured by OD_600_) because it is perhaps the most accessible measure of cellular health and fitness and is an integrator of many other aspects of cell function, thus it may show interesting differences depending on cell history. One can imagine that there might be a strong fitness incentive toward memory in *B. subtilis*. If cells could use a memory of past conditions to ‘predict’ future conditions, and delay sporulation, an expensive process, if the environment is likely to improve or accelerate sporulation if the starvation period is likely to be long, they might improve their odds for long-term survival.

## Results

### Information Theoretic Memory Framework

#### ‘Adaptive’ memory experiment

A complete quantification of biologically relevant memory would involve first perturbing the cell with all possible sequences of complex environmental inputs it might experience in the wild in each of its growth modes, then measuring all cellular responses to these perturbations, and, finally, quantifying the degree and distribution of history-dependence in these responses.

**Figure 2:**
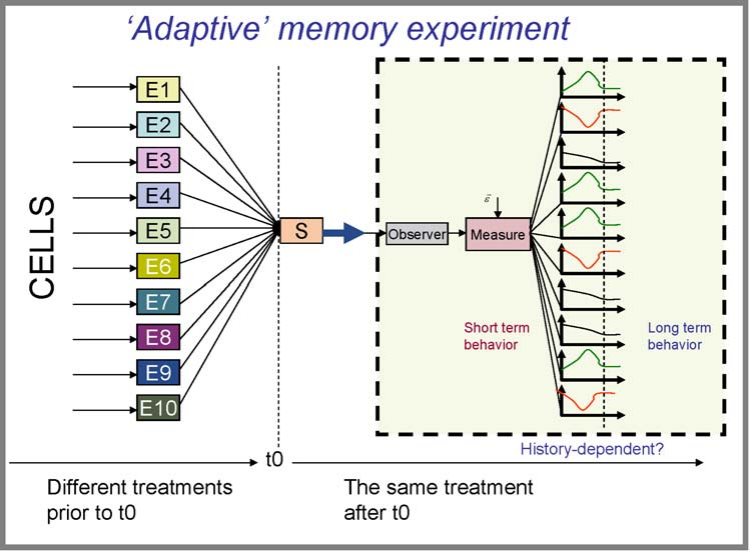
An ‘adaptive’ memory experiment. In an adaptive memory experiment, each (identical) sample of a biological system is subjected to one of several conditions prior to time t0, and then observed in a common condition after t0. If different past histories lead to different short-term behaviors in current conditions, the system can be said to exhibit short-term memory. If different past histories lead to different long-term behaviors, the system can be said to exhibit long-term memory.

Here we assume a simple approximation of this scenario, in which each sample of a biological system is subjected to one of many conditions prior to time t0, and then observed in a common condition after t0 (see Fig. 2 and Definition (1) in Appendix S1 in Supplementary Information). We call this an ‘adaptive’ memory experiment because it roughly simulates a temporal shift in the environment requiring adaptation or acclimation, and to differentiate it from the more classical memory experiments in physics, engineering and cell biology designed to identify hysteretic loops [45]–[47]. While we do not identify such loops here, multistability is suggested by the appearance of long term memory in our experiments. More complex environmental history trajectories could feasibly unravel more memory effects.

We are interested in whether past conditions can be inferred from observations of behavior in current conditions. *The assumption here is that history-dependent behavior is a manifestation of memory, and that the better the possible inference about prior conditions from current measurements, the more memory there is within the system.*

#### Adapting communication metrics to memory

To quantify this intuitive concept of history-dependence as memory, we use concepts from information theory [48] in the tradition of Landauer's use of informational entropy to estimate human memory capacity [49], and the extensive body of work characterizing memory in individual neurons [50]–[53].

**Figure 3:**
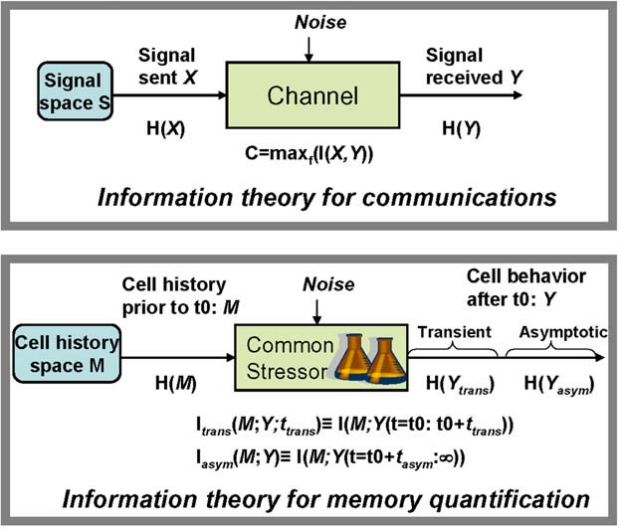
Information-based conceptual schema for measuring memory in microbes. In communication theory (top), the informational entropy of the signal space H(*X*) captures the number of different messages *X* that can be communicated and their probabilistic dispersal; the mutual information I(*X,Y*) between transmitted and received signals quantifies the amount of information actually communicated. A memory experiment, in contrast, involves subjecting cells to distinct treatments *M* prior to time t0, followed by an identical treatment *S* after time t0, with cell behavior from t0 on monitored through temporal sampling of one or more observable variables *Y*. As applied to bacterial memory (bottom), the informational entropy of the cell history space H(*M*) captures the number of different cell histories prior to time t0 tested by the experimental compendium and their probabilistic dispersal; the mutual information I_trans_(*M;Y;t_trans_*) between the transient response of the observable variable *Y* after time t0 and the cell history prior to time t0 captures the short-term memory of cell history exhibited by *Y* over the cell history space in response to treatment *S*. Likewise, the mutual information I_asym_(*M,Y*) between the long-term response of *Y* and cell history prior to t0 captures the long-term memory of cell history exhibited by *Y*.

By interpreting the random variable *Y* as behavior in current conditions, and the random variable *M* as past cellular history prior to time t0, the mutual information *I*(*M*;*Y*) = *H*(*M*)−*H*(*M*|*Y*) of *M* relative to *Y* provides a measure of memory in informational entropy bits (see [48], Fig. 3, and Definition (2) in Appendix S1 for details, including the definition of informational entropy *H*). Roughly speaking, from this perspective *I(M;Y)* captures how much uncertainty about past conditions can be reduced by observations of behavior in current conditions. Worded differently, *I(M;Y)* captures how much information about past conditions can be inferred from observations of behavior in current conditions. *The better the possible inference about prior conditions (and thus the higher the bit count of I(M;Y)), the more memory there is within the system.*

#### Short term vs. long term memory

**Figure 4:**
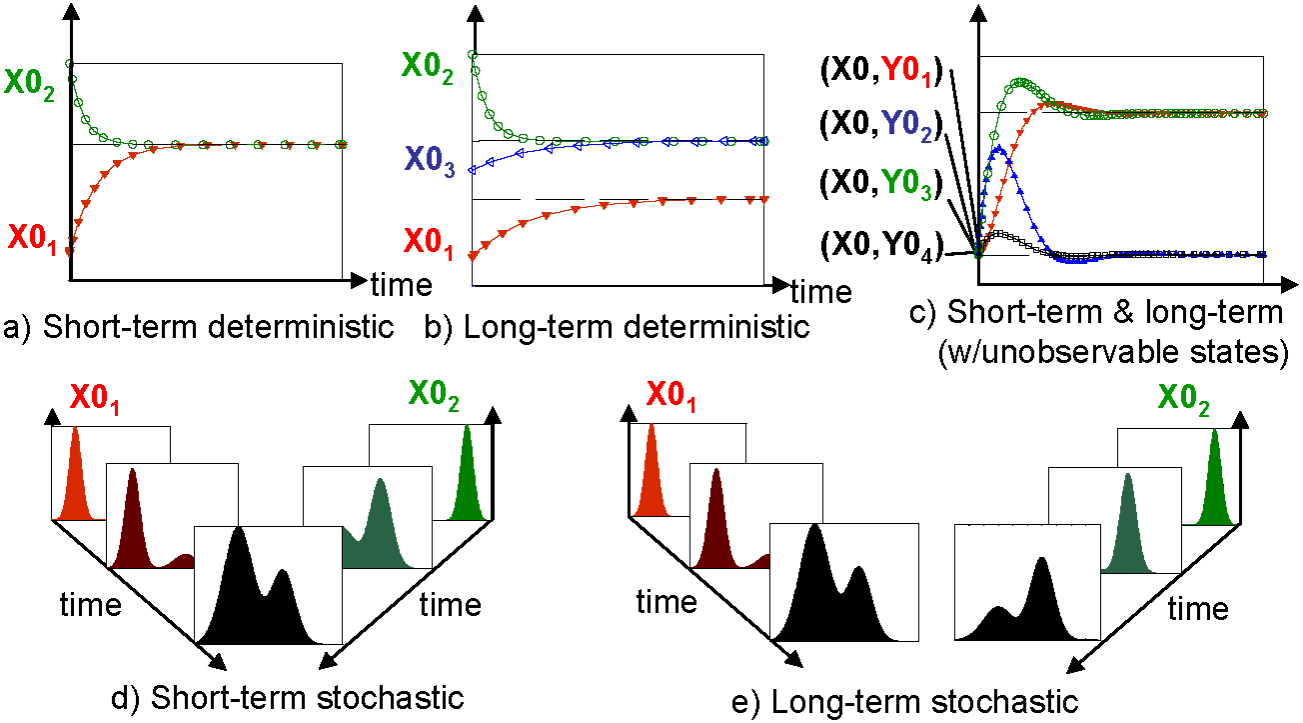
Different types of history-dependent behavior one might observe. a) Short-term deterministic memory. State trajectories ‘remember’ their initial condition for some time, and then converge to a common asymptotic behavior. b) Long-term deterministic memory. State trajectories of multi-stable systems ‘remember’ which basin of attraction their initial condition started in indefinitely (the basin containing X0_1_ vs. the basin containing X0_2_ and X0_3_), but retain a memory of the exact initial condition within a basin of attraction only transiently (X0_2_ vs. X0_3_). c) Short-term and Long-term memory in a system with unobservable states. The state space of the cell is two dimensional (X,Y), but only one of the two dimensions, X, is observed. Though all four initial conditions are distinct in the larger space, the unobserved Y component renders them identical to the observer. Thus the trajectories appear to diverge from a common starting point and approach one of two asymptotic states. This gives the observer the impression of first an increase in information and memory and then a decrease as the trajectories approach their long-term values. d,e) If measurements are made on single cells rather than on averaged populations (as we did in this paper), history-dependent distributions may be observed. d) Short-term stochastic memory. State trajectories are probabilistic in individual cells, with a distribution over the population that initially retains a ‘memory’ of the initial condition of the population. In the long-term, this memory degrades as the distribution approaches a global attractor. e) Long-term stochastic memory. The distribution over the population retains a ‘memory’ of the initial condition indefinitely, or at least over the time-horizon of the experiment.

Memory, or history-dependent behavior, can manifest across multiple time scales. Short term, or transient, memory is stored by the system for some time, and then ‘forgotten’ (see Fig. 4a,d). Systems may also have either ‘effective’ long term memory if the transient dynamics are long compared to environmental fluctuations, or ‘true’ asymptotic memory if the stationary state of the system depends on initial conditions, as occurs in nonlinear systems with multiple attractors (see Figs. 4b,c,and e). For an example of the latter, the state of a bistable switch encodes an asymptotic memory of the last switching event.

Because in many systems the significance, mechanistic origin, and function of memory likely depends on how long it lasts, and in particular whether it can be classified as short-term or long-term, we distinguish between the two types of memory and quantify them separately. From an information perspective, we say that an external observer of an adaptive memory experiment with *a priori* knowledge of the probability distribution over cell histories detects short-term memory in this system if observing measurements of some fraction of the short-term behaviour of the system after time t0 leads to a reduction in uncertainty about the history of the system prior to time t0. In this case, we say that the cells exhibit I_trans_(*M*;*Y*; *t_trans_*)≡I(*M;Y*(t = t0:t0+*t_trans_*)) bits of *short term memory* in the observable *Y* over the period from t0 to t0+trans, where t_trans_ is a time before the signal approaches its steady state (Definition (4) in Appendix S1). Likewise, long-term memory is detected if observing measurements of the system behavior near an apparent steady state after time t0 leads to a reduction in uncertainty about the history of the system. Here we say the cells exhibit I_asym_(*M*;*Y*)≡I(*M;Y*(t = t0+*t_asym_*:∞)) bits of *long term memory* in the observable response *Y* during the experiment, where t_asym_ is the time it takes for the signal to settle (Definition (3) in Appendix S1).

#### Memory quantification normalized

The above metrics for short term and long term memory are absolute measures, in that they give a bit count for an answer. Though these absolute numbers can be useful, it is also useful to measure memory in relative terms, compared to the total amount of memory that *could* be observed in a perfectly retentive system given the limitations of the experiments. To address this issue, we define *short-term memory fidelity* to be P_trans_(*M; Y; t_trans_*)≡I(*M;Y*(t = t0:t0+*t_trans_*))/H(*M*) and l*ong-term memory fidelity* to be P_asym_(*M;Y*)≡I(*M;Y*(t = t0+*t_asym_* :∞))/H(*M*), where H(M) is the entropy over all the past conditions that were applied in the experiment. These normalized mutual information metrics, measures between 0 and 1 of the fraction of uncertainty about the past conditions tested that is reduced by knowledge of future cellular response, have also been called the coefficients of constraint [54] (see Definition (5) in Appendix S1).

#### Quantifying memory in higher dimensions

In addition to analyzing each observable individually, we are interested in calculating the short and long term memory exhibited by the combined behavior of multiple observables. To do so, the above definitions are easily extended to the case of multiple observables by letting Y be a vector *Ȳ* = (*Y*_1_,…,*Y_n_*) and calculating I_asym_(*M*;(*Y_1_,…,Y_n_*)) and I_trans_(*M*;(*Y_1_,…,Y_n_*); *t_trans_*) and the memory fidelity of each. This combined-memory estimation is interesting because it allows one to address the question of whether combining information from multiple read-outs leads to extra memory beyond what is present in any of the individual read-outs, and if so, how much. This issue is related to the size of the memory, and the dimension it occupies within a cell's state space.

An inequality governing the informational entropy of a vector pair of variables (X,Y) is as follows: max(*H*(*X*),*H*(*Y*))≤*H*(*X*,*Y*)≤*H*(*X*)+*H*(*Y*) [54]. Thus, we know that the memory exhibited by any pair of observables must be greater or equal to the bit count of the most retentive pathway of the pair, and less than or equal to the sum of the bit counts of the two pathways. If two pathways are controlled independently, their combined behavior could produce the upper limit on memory in the higher-dimensional space, whereas if the pathways are controlled by a common signal or if one pathway hierarchically controls the other, the lower limit might be realized. To quantify this concept, we define *memory orthogonality* between two pathway readouts *Y1* and *Y2* to be: *Mem_orth_(M;(Y1,Y2)) ≡ (I(M;(Y1,Y2))-max(I(M;Y1),I(M;Y2)))/min(I(M;Y1),I(M;Y2))*, where *M* is cell history and *I* is mutual information. *Mem_orth_* equals 1 if the two variables combined as a vector yield the upper bound of memory, and 0 if the two variables in combination yield the lower bound (see Definition (6) in Appendix S1).

#### Implementation

For the calculations above, listed more formally in Appendix S1 in Supplemental Information, we need to estimate probability distributions over the past cell histories being tested and the responses of the cells to each history. For past conditions/histories, we enforce a uniform probability of observation of each condition by running each experiment (condition i = > response i) a fixed number of times. For responses, we cluster trajectories from the different conditions and the probability of a response is simply the histogram of trajectories over clusters. The probability of prior environment given cluster membership is enumerated in a similar way. Details of the entire analysis algorithm can be found in Materials and Methods.

#### Caveats

The above information-based metrics and simple associated analysis algorithm (see Materials and Methods) are useful in that they transform the ‘lay’ questions–“Do cells ‘remember’ past experiences and use these memories to modify future stress response dynamics?” and “If so, is this ‘memory’ short term or long term, and how much is there?”–into well-defined queries about information and uncertainty yielding quantitative estimates of microbial memory in informational entropy *bits*.

However, any attempt to quantify or qualify memory is fundamentally limited by the possibility of unobservable states (see Fig. 4c), uncontrolled and unobservable inputs, poor choice of input combinations and sequences, and measurement errors and distortions. Here we assume most such limitations, discussed in more detail in Supplementary Information (Section S1), are inherent in the estimation of memory processes and most likely to result in information loss and thus *underestimates* of the ability of the system to ‘remember’ the cell histories tested by the experimental compendium. Therefore we interpret quantifications of memory within our *B. subtilis* compendium as lower bound estimates.

### Experiment and Overview of Analysis

Memory experiment on *B. subtilis*: To test for history dependent behavior–‘memory’-in *B. subtilis*, we engineered a fluorescently labeled strain of *Bacillus subtilis* to report on commitment to sporulation and degradative enzyme synthesis: the KEE strain (P*spoIIE-gfp*, P*aprE-dsred cmp*, see Materials and Methods for details on strain construction). The *spoIIE* promoter (P*spoIIE*), our sporulation reporter, controls expression of *spoIIE*, a gene encoding a serine phosphatase specifically expressed upon commitment to sporulation and therefore considered a good sporulation commitment signal [55], [56]. The *aprE* promoter (P*aprE*), our degradative enzyme synthesis reporter, controls expression of the extracellular protease subtilisin naturally produced by *B. subtilis* cells at the end of exponential growth [57].

With the KEE reporter strain, we used our framework to estimate, in informational entropy bits, the capacity of these stress response pathways and of the cell growth dynamics to ‘remember’ 10 distinct cell histories prior to application of a common stressor. Specifically, we first grew three replicate cultures in one of two media, Luria Broth medium (LB) or growth medium (GM) [58], to one of five different densities (all still in exponential growth, ranging from OD_600_ = [0.1:1], see Table 1, where OD_600_ is the optical density of the culture at 600nM), for a total of ten cell histories. Thus in the first stage of the experiment, a clonal population of cells was divided into 30 groups, each of which experienced one of the 10 cell histories consisting of growth in one of two media to one of five cell densities over a fixed period of time (see Materials and Methods for details).

**Table 1:** Cell history table.

n	Cell history	Cell History Description
1	LB: D	Grown in LB (rich medium) to density D (OD_600_ = 1)
2	LB: −1	Grown in LB to density −1 (OD_600_≈0.65)
3	LB: −2	Grown in LB to density −2 (OD_600_≈0.4)
4	LB: −3	Grown in LB to density −3 (OD_600_≈0.2)
5	LB: −4	Grown in LB to density −4 (OD_600_≈0.1)
6	GM: D	Grown in GM (less rich medium) to density D
7	GM: −1	Grown in GM to density −1
8	GM: −2	Grown in GM to density −2
9	GM: −3	Grown in GM to density −3
10	GM: −4	Grown in GM to density −4

The cell history space M consists of 10 cell histories *M* = (*Medium1,Density1*): growth in either rich Luria Broth medium (LB) or a less rich growth medium (GM) [58] to one of five cell densities, D, −1, −2, −3, −4.

We chose to combine different media with growth to different densities as our set of cell histories because growth media can impact cell state, as can growth of cultures to different densities over a fixed period of time. Cells deplete nutrients and respond to the environment and its dynamics with changes in metabolic fluxes, post-translational modifications, gene expression, quorum signaling and synthesis of storage compounds. GM medium (also called CH medium) is a rich medium with casein hydrolysate as the sole carbon source [58]. LB medium is a much richer and more complex medium than GM and therefore sustains more rapid growth. We assumed that any resulting history-dependent differences in cell state at time t0 might lead to different history-dependent behaviors in the common medium after t0.

After experiencing one of the 10 different cell histories, cells were then pelleted and resuspended at an intermediate density (OD_600_ = 0.5) in a common stress medium, in this case, sporulation salts starvation medium (SM) [58]. The resuspension time is denoted t0. Thus, regardless of past experiences, all cells observed after t0 were subjected to starvation conditions starting at t0 in a fixed-density, fixed-size population.

**Figure 5:**
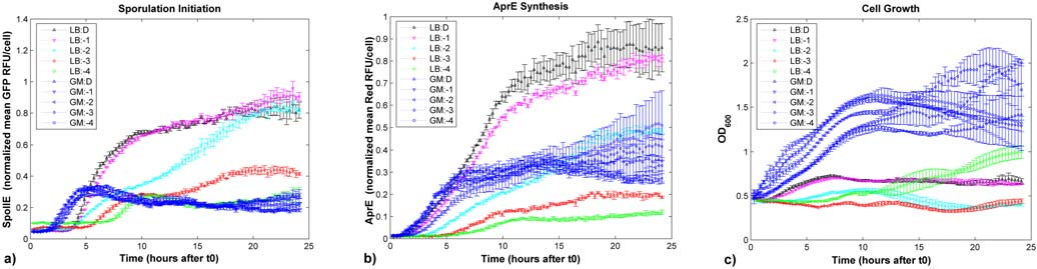
*B. subtilis* memory data compendium. These plots show the dynamics of the sporulation initiation reporter P*spoIIE-gfp expression* (a), the degradative enzyme synthesis reporter P*aprE-dsred* expression (b), and cell growth (c) of *B. subtilis* KEE after the onset of starvation (resuspension in SM) as a function of cell history prior to starvation, as measured by fluorescence (GFP, and DsRed) and OD_600_ time series measurements taken every 15 minutes for 24 hours, respectively. The 10 cell histories tested consisted of growth in either rich LB medium or poorer GM medium to one of five densities D, −1, −2, −3, −4, (see experimental overview section and Materials and Methods for details). Fluorescent intensities in (a–b) were divided by OD600 (c) and then normalized to a [0 1] scale by dividing by the maximum. The error bars show standard deviation over replicates at each time point.

Our three observables *Y* after t0 consisted of two fluorescent reporters, one for sporulation initiation and another for degradative enzyme synthesis (strain KEE (P*spoIIE-gfp*, P*aprE-dsred cmp*)), and optical density of the culture as a proxy for cell growth (OD_600_), measured at the bulk population level every 15 minutes for 24 hours starting at time t0 (see Fig. 5 for time series, and Materials and Methods for details on strain construction and experiments). Thus, with 30 cultures–three for each of the 10 cell histories– and three observables per culture measured every 15 minutes for 24 hours in the common stress medium starting at t0, the memory data compendium for this set of experiments consists of 30×3×96 = 8,640 measurements arranged in a 90 by 96 matrix.

Data analysis overview: The resulting memory data compendium was then analyzed for short- and long-term memory in each output signal individually and in all possible combinations of the three signals by applying the memory quantification algorithm described in detail in Materials and Methods and illustrated in the flow chart in Supplementary Information Section S2.

To briefly summarize, in order to estimate how much short-term and long-term memory was manifested in the behavior of the reporters, we sought to calculate the mutual information between the behavior of the cells after t0 and the history of the cells before t0. This calculation required that we estimate the joint probability density between cellular behavior after t0 and cell history prior to t0. Given constraints on the amount of data and other considerations described in detail in Section S3 of Supplementary Information, we took a clustering approach to this problem. That is, we first clustered the response of the pathway reporter as a way of dividing the trajectories into groups with common, distinct behaviors. The resulting assignment of each trajectory to a cluster was then used to calculate the frequency of co-occurrence of each behavioral class and each possible cell history. From this histogram we estimated the requisite joint probability distribution, which was then used to calculate the mutual information between cell history and the behavior of the observable, and thus arrive at an estimate for memory.

We performed this procedure on the 30 trajectories (3 replicates for each of the 10 cell histories tested) of each of the three observables, using both the short term (first 11 hours of measurements, during which the signal was still dynamically varying-see Materials and Methods for more details on our choice of analysis intervals) and long-term response (last three hours of measurements, from 21 to 24 hours, by which time the signals have remained flat for several hours) in order to estimate short-term and long-term memories manifested in each individual signal. To calculate the short-term and long-term memory in the combined activities of multiple signals, we took the same approach, with the one difference being that the clustering step captured the combined behavior of multiple readouts (Step 3 in the algorithm in Materials and Methods). All bit counts were then normalized to calculate memory fidelities and orthogonalities, as defined in Appendix S1, in order to estimate in relative terms how much of the total possible memory each system ‘remembers’, and how much ‘extra’ memory is embedded in the higher-dimensional spaces formed by multiple pathways.

Since the 30 populations were subjected to 10 different (within error) past conditions *M* = (*Medium1, Density1*) in equal proportions, the informational entropy of the cell history space M is H(*M*) = −log_2_(1/10) = 3.3219 bits. Thus, without prior knowledge there are 3.3219 bits of information about cell history at most that can be recovered from observation of these three outputs, either individually or in combination and on any time scale.

### Experimental Results

#### A qualitative overview of history-dependence

The *B. subtilis* stress responses measured by the three observables (Figure 5) appear neither memoryless nor in possession of a perfect memory of the cell histories tested. They do not appear to be memoryless because not all signals from a given observable follow a common trajectory (within noise bounds) irrespective of past history of the cells. Nor does the memory of any observable appear to be perfect, because though there are ten distinct cell histories prior to time t0, there appear to be fewer than ten distinct dynamics per observable in response to the starvation stressor administered at time t0. By eye, there appear to be more distinct behaviors in the short term than in the long term. Also, different cell histories group together for different observables. This means that we expect a higher bit count estimate of short term memory than long term memory, and different amounts of memory and of different aspects of cell history in the three pathway observables.

#### All observables exhibit short-term memory of cell history, with sporulation exhibiting the most and growth dynamics the least

**Figure 6:**
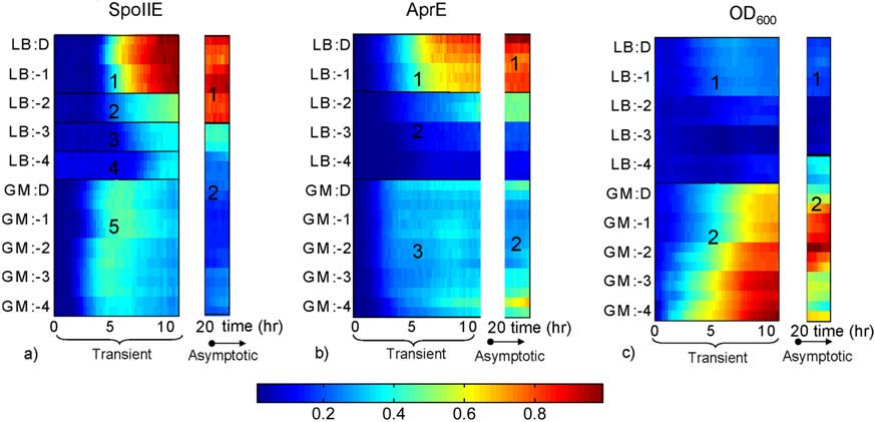
The map from cell history to *B. subtilis* stress response clusters. The transient dynamics and long-term levels of the sporulation initiation (P*spoIIE-gfp* expression), AprE synthesis (P*aprE-dsred* expression), and growth (OD_600_) signals were clustered using the automatic method in Materials and Methods. This figure shows the heat maps for each signal in Figure 5 (dark red indicates maximum, and dark blue minimum), the number of behavioral classes for each signal, and which subset of the ten cell histories in our test set corresponds to each cluster. For example, the asymptotic sporulation initiation signal from P*spoIIE*-*gfp* fusion clustered into two classes, one (top, 1) corresponding to a history of growth in rich LB medium to the three highest densities, D, −1, and −2, and the other class (bottom, 2) corresponding to all other cell histories.

The transient behavior (first 11 hours) of the SpoIIE (sporulation) reporter clusters into five distinct classes of behavior (different onset times and sigmoidal vs. more pulsatile expression), whereas the transient behavior of the AprE (degradative enzyme synthesis) reporter clusters into three classes (different onset times and different expression levels) and the growth reporter into just two classes (some vs. almost no growth) (see left panels of Fig. 6a,b,c). The mutual information between the resulting clustering vectors and the cell history vector captures how well the different behavioral classes of each observable correspond to different cell histories. Performing this calculation, we estimate I_trans(spo)_ = *1.96 bits* of short-term memory in the sporulation reporter; I_trans(AprE)_ = *1.4855 bits* of short-term memory in the degradative enzyme synthesis reporter, and I_trans(OD)_ = *1 bit* of short-term memory in the growth dynamics reporter OD_600_. Thus, all three observables exhibit short-term memory of the cell histories tested, with the sporulation reporter exhibiting the most memory and growth dynamics the least.

**Figure 7:**
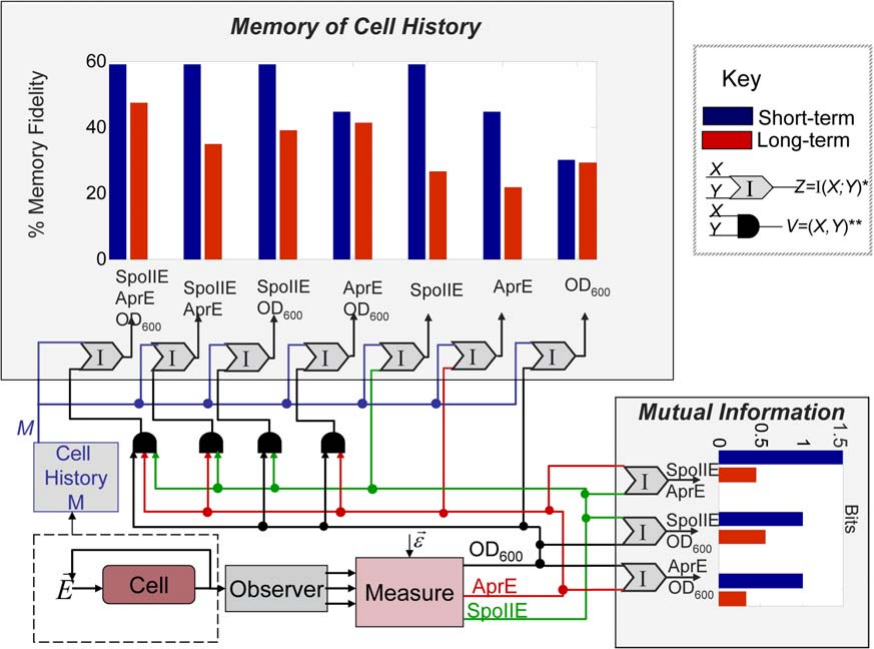
Estimates of cell-history memory and mutual information in *B. subtilis.* The upper left bar plot shows our estimate of long-term (blue bars) and short-term (first 11 hours, red bars) memory fidelity (% of the maximum recoverable information about cell history) exhibited in starvation medium SM by sporulation initiation (P*spoIIE-gfp* expression), degradative enzyme synthesis (P*aprE-dsred* expression), and growth dynamics (OD_600_), and over all vector pairs of observable read-outs and the vector triple, with respect to the cell history space tested by our compendium. The lower right bar plot shows our estimate of the number of bits of mutual information shared by all pairs of short-term (red bars) and long-term (blue bars) observable signals in our memory data compendium. The surrounding flow diagram circuit illustrates the experimental and analytical scenario.

Dividing these absolute bit counts by the entropy of the cell history space, we estimate the short-term memory fidelities of sporulation initiation, degradative enzyme synthesis, and growth dynamics to be P_trans(spo)_ = I_trans(spo)_/H(M) = 1.96/3.3219 = 0.59, P_trans(AprE)_ = I_trans(AprE)_/H(M) = 1.48/3.3219 = 0.45, and P_trans(OD)_ = I_trans(OD)_/H(*M*) = 1/3.32≈0.3, respectively. This means that if one were to observe all 30 short-term responses of one of the three reporters after t0 but not told which history corresponds to which trajectory, 59% of the uncertainty about cell history prior to time t0 could be reduced by observation of the transient sporulation reporter dynamics after time t0, 45% of this uncertainty about the past could be reduced by observation of the degradative enzyme synthesis reporter dynamics after t0, and only 30% of this uncertainty could be reduced by observation of the growth dynamics after t0. More intuitively, one could say that 59%, 45% and 30% of the cell histories tested are ‘remembered’ by the short-term dynamics of the sporulation, degradative enzyme synthesis, and growth reporters, respectively (see Fig. 7 and Table S1).

#### All observables exhibit long-term memory of cell history, though at a lower bit count than short-term memory

Though short term memory can be important—because even short term behavioral differences may have fitness consequences [59], especially if they are on the order of environmental fluctuations [28], [60]—long term memory is generally the first thing that comes to mind when memory is discussed [61]–[64]. One might expect long term memory in *B. subtilis* stress responses-sporulation control especially-because of the feedback topologies in their regulatory circuitry and reportedly bistable behaviors [10], [36]–[39].

To estimate the long term memory in each individual pathway we first clustered the final segment of the 30 time series of each reporter (from 21 to 24 hours after t0) to estimate the number of distinct long-term behaviors for each of the three pathway reporters (results = *2* unequal-sized clusters for each reporter, as shown in Fig. 6, though the cluster sizes and associated cell histories differ across reporters). We then calculated the mutual information between the clustering results and the cell history vector to arrive at lower bound estimates of I_asym(spo)_ = *0.8813 bits,* I_asym(AprE)_ = *0.72 bits*, and I_asym(OD)_ = *0.97 bits* of long-term memory in the networks controlling sporulation initiation, AprE synthesis and growth dynamics, respectively. Thus, like a switch, there appear to be two, stable, long term behaviors for each pathway reporter, though the probability of converging to each is not equal or the same across reporters, as is reflected by distinct bit counts of less than 1 (if half the past histories lead to one attractor and the other half of the histories lead to the other, there would be 1 bit of asymptotic memory).

Dividing these absolute numbers by the entropy of the cell history space, we estimate the long-term memory fidelities of sporulation initiation, degradative enzyme synthesis, and growth to be P_asym(spo)_ = I_asym(spo)_/H(*M*) = 0.8813/3.3219 = 0.265, P_asym(AprE)_ = 0.22, and P_asym(OD)_ = 0.29, respectively. Thus, approximately 25% of the uncertainty about cell history prior to the onset of starvation is reduced by knowledge of any one of the three long-term reporter dynamics in the starvation environment. To summarize, all three observables exhibit around 1 bit of long-term memory of the histories tested, though of different aspects of cell history as will be shown below. One bit is a significant amount but much less than the nearly 2 bits of memory seen in the most retentive short-term response.

#### Different observables remember different aspects of cell history to different degrees

The above memory estimates are in a sense high-level, because each of the 10 distinct cell histories is treated identically. By drilling down a level of resolution to the component parts of the cell histories–initial nutrient composition of the media and cell density reached in that media (which can also feasibly affect both the nutritional composition of the medium and cell state while in log phase)–we can investigate which aspects of cell history are remembered by the observables and for how long.

In the short term, all three observables have a perfect memory of whether they were grown in LB or GM, and only a partial memory of their density in this medium. Put more formally, if we consider growth medium in isolation and calculate the mutual information between growth medium prior to time t0 and transient response of the three reporters to starvation after time t0, we see that a history of growth in LB can be distinguished from a history of growth in GM with 100% memory fidelity (P_trans(Spo)_(*Medium1*;*Y*;*t_tran_*_s_ = 11 hrs) = P_trans(AprE)_(*Medium1*;*Y*;*t_tran_*_s_ = 11 hrs) = P_trans(OD)_(*Medium1*;*Y*; *t_tran_*_s_ = 11 hrs) = 1, where *Medium1* is a random variable representing growth medium prior to time t0, and can take on the values GM or LB). In contrast, the ability of the pathways to remember the population density reached prior to t0 (and any changes in cell state these differences in cell density create) is less simple. With a history of growth in GM, the cell density prior to the onset of starvation at t0 is not ‘remembered’ by the short-term *B. subtilis* sporulation, degradative enzyme synthesis, or growth dynamics responses, even transiently (0% memory fidelity), as all responses are indistinguishable within noise (I_trans_(*Density1/Medium1* = GM;*Y*; *t_tran_*_s_ = 11hrs) = 0). However, when grown in LB, the cell density prior to t0 is remembered with 80% memory fidelity by the transient sporulation dynamics and with 60% memory fidelity by the transient AprE dynamics (P_trans(Spo)_(*Density1*/*Medium1* = LB;*Y*; *t_tran_*_s_ = 11 hrs) = 0.8; P_trans(AprE)_(*Density1*/*Medium1* = LB;*Y*; *t_tran_*_s_ = 11 hrs) = 0.6).

In the long term, all three observables have only a partial memory of which medium they were grown in, and to what density. Like in the transient memory case, past growth medium is remembered better than past cell density, but unlike in the transient memory case, there is no perfectly clean dividing line separating out the long-term responses to the two growth media histories. For example, given observations of the long-term behavior of the sporulation reporter, a history of growth in LB can be distinguished from a history of growth in GM with only *39% memory fidelity* (I_asym(spo)_(*Medium1*;*Y*)/H(*Medium1*) = 0.39), whereas cell densities (grouped into five classes, (D,-1,-2,-3 and -4)) prior to t0 are remembered even less well, with only 12.1% memory fidelity (I_asym(spo)_(*Density1*;*Y*)/H(*Density1*) = 0.121). A similar pattern can be seen in the long-term memories of the other two reporters. Interestingly, though each reporter exhibits two possible long-term behaviors, the clusters are different sizes and the histories that correspond to each behavioral cluster are different for different pathways. As will be shown in the next section, these differences lead to the possibility of an increased memory capacity in the higher dimensional space defined by the combined activities of multiple pathways.

#### There is more long-term memory in the combined activity of the observables than is present in any individual observable

Interestingly, analysis of the *transient memory* of the pairs of pathway readouts (Spo, AprE), (Spo, OD_600_), and (AprE, OD_600_)) shows *no increase in memory in the higher dimensional space* than is found in the most retentive pathway in the dyad (see Figure 7). For example, we estimate the transient memory found in the pair (AprE, OD_600_) to be 1.4855 bits, which is the same bit count found in AprE alone (*Mem_orth(trans)_*(*M*;(*AprE, OD_600_*)) = (1.4855-1.4855)/(1.4855) = 0). Likewise, the three-dimensional readout (Spo, AprE, OD_600_) shows no more transient memory than is found in the sporulation pathway (1.96 bits), its most retentive member.

However, the same conclusion does not follow for asymptotic memory. *Every pair of pathway readouts contains more asymptotic memory than either constituent signal, and the triple pathway readout contains more asymptotic memory (at 1.57 bits) than any of the constituent pairs* (see Figure 7). This implies that the long term behavior of our three observables occupies a relatively high dimensional space, with each subsystem responding differently to aspects of past conditions. For example, though the AprE pathway is estimated to have only 0.7219 bits of asymptotic memory and the growth measure OD_600_ has only 0.971 bits of asymptotic memory, the pair (AprE, OD_600_) has 1.371 bits of asymptotic memory (*Mem_orth(asym)_*(*M*;(*AprE*, *OD_600_*)) = (1.371-0.971)/(0.7219) = 0.554, or 55.4% of the maximum). Put more concretely, the asymptotic behavior of the AprE signal alone ‘remembers’ two classes of cell history: the first a history of growth in rich medium to higher densities and the second all other histories in the compendium. Whereas observations of the asymptotic behavior of the growth signal allow distinction between two *different* classes of cell history; the first growth in rich medium to all densities greater than the lowest tested (-4), and the second all other histories in the compendium. Viewed together as a combined vector in a higher dimensional space, the asymptotes of the pair (AprE, OD_600_) permit distinction between three classes of cell history: growth in rich medium to higher cell densities, growth in rich medium to low (but not lowest) and intermediate cell densities, and, finally, growth in rich medium to the lowest density or growth in poorer medium to any density. Adding the sporulation signal increases the information storage yet again, by adding another discernable class, leading to a total long-term combinatorial storage of 1.57 bits. Thus, because the different cellular systems in *B. subtilis* remember different aspects of prior history, the combined activity of multiple pathways is able to combinatorially store more information about the past than can any individual pathway. However, the total asymptotic memory is still somewhat less than the total transient memory (1.57 vs. 1.96 bits). (For a complete accounting of cell history memory over all signal combinations, and for the mutual information between all pairs of signals, including the transient and asymptotic responses of each signal, see Figure 7 and Table S1.)

## Discussion

Though evidence that bacterial cells are able to remember their histories and use these memories to alter their behavior in a fitness enhancing manner would not raise expectations that bacteria could recite π or write music, it *would* enrich the motifs-modules-games view of bacterial regulation [12] by adding game strategies *with memory* to the repertoire of microbes. This exploratory paper does not provide evidence that *B. subtilis*, or any other microbe, is intelligent or is playing an evolved, fitness-enhancing memory strategy. Rather, in this work we propose that the familiar phenomenon of history-dependent behavior in microbes reflects a form of memory worth studying systematically and quantifying, and that doing so sets the foundation for understanding both the mechanisms and function of memory in cell behavior and fitness. To this end we formulated a conceptual information-theory based framework for measuring microbial memory, thereby introducing tools that begin to observe and quantify the relationship between past cell history and future cell behavior from a new angle. This method produces a phenomenological measure of cellular memory without regard to the specific cellular mechanisms encoding it.

We then applied these tools to a simple set of medium-shift experiments on *B. subtilis*, in the process demonstrating that *B. subtilis* does ‘remember’, both in the short and long term, aspects of its cell history, and that this memory is distributed differently among the observables. More short term than long term memory was evident, with short-term sporulation dynamics exhibiting the most memory and long-term degradative enzyme AprE synthesis dynamics the least. As expected, some but not all of the history-dependence between the sporulation and AprE reporters is shared (AprE has 75% of the short-term and 80% of the long-term memory estimated for sporulation). We also illustrated how to quantify memory in multiple combined variables, in the process showing that because the different cellular systems in *B. subtilis* remember different aspects of prior history to different degrees, the combined activity of multiple pathways is able to combinatorially store more information about the past than can any individual pathway. Of the two components of cell history varied in our compendium–past growth medium and the cell density reached in this medium, which can alter cell state even in log phase–growth medium appeared to be better remembered by *B. subtilis*, with past density remembered best when originally grown in the medium richest with nutrients, LB. Admittedly we do not yet know whether the memory we have observed is fitness enhancing and evolved or just incidental, or what molecular mechanisms or artifacts are responsible for the observed pattern of memory storage. Rather, these simple experiments and the surrounding analysis and framework demonstrate what could be the beginning of a larger memory program, and indicate that memory in cellular behaviors may be a rich area for further exploration.

### Ideas for a more complete memory-in-microbes research program

A more complete program for investigating memory in bacteria would encompass at least three lines of inquiry, essentially the ‘what’, ‘how’, and ‘why’ of bacterial memory. The first line of inquiry (what), for which this study is an example, is the quantification of environmental memory in a microbe. This study could be extended by resolving the population-averaged behavior analyzed in this paper into single-cell measurements and memory classification and quantification. Given that sporulation is thought to be a stochastically triggered bistable developmental process [10], [36]–[39], one might expect the population-averaged measurements (Figure 5.b) to resolve into bimodal distributions of high and low GFP-expressing cells. And since AprE synthesis control is believed to be more deterministic and analog, one might expect more monomodal distributions. Preliminary data from flow cytometry analysis support this expectation, at least for some histories and time points (see Figure S1 in Supplemental Information), but further work is needed to determine for what conditions and pathways memory at the single-cell level can be classified as stochastic, and the form and quantification of this stochasticity. An exploration of the memory characteristics of other cellular players active in these and interacting networks, and the space of their environmental sensitivity, with the goal of estimating the ‘true’ memory capacity of the system, are other possible extensions of this work.

A second line of inquiry (how) would build upon the first by elucidating the causal basis for any observed environmental memory. Though many genetic and epigenetic bacterial switching mechanisms have been elucidated [8], [10], [16], still unclear is exactly how different types of environmental and intercellular signals might be encoded and remembered within cellular circuitry for varying lengths of time, a question addressable through mutant studies and modeling. On the ‘meta’ level one could ask whether memory is stored within single cells, population distributions, or in the larger state space defined by the cell-environment interaction through distributions of nutrients, waste products, enzymes, signaling molecules, biofilm generating conditions, and so on. A third line of inquiry could focus on the ‘why’s' of environmental memory. Is environmental memory, if it exists, controlled or incidental: evolutionarily advantageous, deleterious, or neutral? Is there evidence that memory-modulation of phenotype expression control does not provide a fitness advantage in the *present* but rather in a *future* implicitly anticipated from past experiences, thus implying an internal model of environmental dynamics (in analogy to the internal model principle in control [65])? We suspect that answers to these ‘why’ questions could be key to whether the others are worth deeply pursuing.

### What do the *B. subtilis* memory observations in this case study mean?

Though we do not yet know whether the memory we have observed is fitness enhancing and evolved, or merely incidental, we can speculate. Looking qualitatively at the three behavioral observables together, we notice that when cells are grown to low density in the less rich GM medium prior to the onset of starvation conditions, they on average grow very fast after resuspension in starvation media, and after a brief lag start turning on their degradative enzyme synthesis and their probabilistic sporulation machinery, even as the population continues to grow. Whereas when cells are grown in richer, nutrient filled LB medium to the same low density prior to the onset of starvation conditions, they take a quite different approach. In this case, cells seem to adopt a wait-and-see strategy, forgoing growth and delaying sporulation and AprE synthesis for many hours.

A game strategy with memory?: The most tempting speculation is that *B. subtilis* is playing a memory strategy in an evolutionary game. From a game perspective, one could take these observations as a sign that after transitioning from a less rich medium to starvation, *B. subtilis* uses its memory of past nutrient-limited growth in the context of an implicit internal model of environmental dynamics to ‘predict’ how long starvation conditions will last. If the cells expect starvation to last a long time, a rational course of action might be to create as many spores as possible, as fast as possible, to maximize the spore count that will lie dormant until the next period of nutritional plenty. On the other hand, if growth in a rich environment prior to starvation in the context of this internal model produces a prediction of a short period of starvation, the rational action might be to *delay* sporulation, thereby decreasing the chances of having committed irreversibly to an unnecessary, costly 8 hour developmental program during which conditions could improve and the cells could be growing. Viewed in this way, *B. subtilis*'s cell-history dependent behavior might constitute an evolved probabilistic memory strategy in its game of survival. Such a strategy would trump diversification strategies without memory [28]–[30], [35], [66], [67], and be analogous to adaptive model-based bet hedging over a diversified portfolio in the stock market [28].

If the above scenario is true, one would expect sporulation initiation *delay* to be a likely feature of the sporulation regulation strategy of *B. subtilis* to exhibit memory. Within our experimental compendium, the delay in turning on the sporulation machinery, as estimated by the amount of time it takes for GFP to start being noticeably expressed from the SpoIIE promoter by a population (normalized GFP intensity >0.035, after which GFP rapidly increases), ranges from a relatively short 1.5 hours to a much longer nearly 8 or more hours after the onset of starvation (Figs. 5b and S2 in Supplemental Information). Calculating the mutual information between GFP expression delays and cell history, we see that most (86%) of the short-term memory in the sporulation reporter can be recapitulated by reducing the trajectories to this single number (I(*M; Initiation Delay*)/I_trans_(*M;Y;t_trans_* = 11hrs) = 1.685/1.96 ≈ 0.86). This calculation does not prove that the history-dependence we have observed is an evolved and fitness enhancing memory strategy in a game, but it is suggestive.

…or an artifact of metabolism?: Then again, the explanation could have little to do with evolutionary games. It could be that differences in metabolic stores, housekeeping apparatus, or metabolic state induced by the different media and different biomass of the culture simply represent initial conditions from which entry into sporulation and other stress responses is more or less easy [1]. For example there might be more ribosomes after growth in LB than there are after growth in GM, forcing cells coming from the latter to stop growth and initiate sporulation sooner. Or it could be that growth in GM, a medium that while not nutrient-limited is lacking the excess of simple carbon and nitrogen sources and readily available amino acids found in LB, activates metabolic pathways that can facilitate growth and spore formation in stress conditions. Then, when transferred to starvation conditions, cells might be able to use this metabolic machinery (and perhaps some form of intracellular nutrient storage) to scavenge whatever scarce nutrients are to be found in the new medium in order to grow and turn on their sporulation and degradative enzyme pathways nearly immediately. Whereas with a history of growth in rich, complex LB medium, cells might enter starvation conditions of SM without enzymatic or other reserves necessary for a near-immediate response to severely limited conditions, and thus require a delay while the cells construct the necessary metabolic machinery to acclimate to their environment.

These possibilities are not mutually exclusive; history-dependent behaviors could stem from some combination of evolved diversification game strategy and artifactual adaptive metabolic processes. Experiments comparing the fitness of wildtype bacteria to mutants with disrupted memory mechanisms coupled to a game theoretic analysis will be necessary to distinguish among the possibilities, and would identify the mechanistic source of memory behaviors in the process. In any case, we hope that this conceptual framework and analytical approach to quantifying memory in cellular behaviors will be a useful point of departure for studying a new set of questions about cellular regulation and evolutionary strategy in microbes.

## Materials and Methods

### Strains and culture media

*Bacillus subtilis* 168 *trpC* (Bacillus Genetic Stock center) was used as the wild-type strain. *Escherichia coli* strain DH5α was used for all plasmid amplifications and isolations. *Escherichia coli* was grown at 37°C in LB supplemented, when necessary, with ampicillin at a final concentration of 100 µg/ml. *B. subtilis* was cultured at 37°C in either LB, growth medium (GM) or sporulation medium (SM). GM and SM media are commonly used in the ‘induction of sporulation by resuspension protocol’ described by Harwood and Cutting [58] and were supplemented with 50 µg/ml and 20 µg/ml L-tryptophan respectively. Antibiotics were added, with the following final concentrations: chloramphenicol, 5 µg/ml; spectinomycin, 100 µg/ml.

### DNA isolation and manipulation

Total genomic DNA from *B. subtilis* 168 was isolated with DNeasy Blood & Tissue Kit (Qiagen) following manufacturer's protocol for Gram positive bacteria. Plasmid DNA was extracted from *E. coli* with the QIAprep kit (Qiagen). DNA restriction and cloning were performed according to standard procedures [68]. Restriction enzymes and T4 DNA ligase were obtained from New England BioLabs and used according to the manufacturer's instructions. DNA fragments were purified from agarose gels with the QIAquick gel purification kit (Qiagen). *Vent* DNA polymerase (New England Biolabs) was used for PCRs.

### *B. subtilis* reporter strain construction

Strains and plasmids are listed in Table S2 in Supplemental Information. To integrate the fluorescent reporter fusions in the *B. subtilis* genome the pLFKEE integration vector was constructed as followed. The GFP variant GFPmut2 [69] was excised from pMF19 [70] by digestion with *BamHI/EcoRI* enzymes and ligated into pEA18 (a gift from Antje Hofmeister) digested with the same enzymes, to give pLF22. The plasmid pEA18 (*cmp*, *spc*) is a vector [71] allowing integration by double cross-over at the *amyE* locus, with a chloramphenicol selection. The *spoIIE* promoter (P*_spoIIE_*) was amplified by PCR from *B. subtilis* 168 genomic DNA using primers PspoIIE-D/*EcoRI* (atcacggaattcaaatcggtttctcttgcagaagccg) and PspoIIEM-R/*HindIII* (atacaaagcttttatattcgttgcctgtcattatagcg), and digested with *EcoRI and HindIII*, then ligated 5′ of *gfpmut2* on pLF22 that had been digested with the same enzymes to give pLF25 (P*_spoIIE_*-*gfp*, *cmp*). The transcriptional profile of the *spoIIE* gene was verified by total RNA dot blot before and after induction of sporulation to confirm its early and specific expression induction at the onset of sporulation (see Figure S3).

To obtain the P*_aprE_*-*dsred* fusion, the *dsredexpress* coding sequence was amplified by PCR from pDsRed-Express (Clontech) using primers DsRed-D/*FseI* (tacggccggcctaaggaggaactacaaatggcgagcagtgaggacatcatcaagg) and DsRed-X/*EcoRV* (agatatcgatcagatctacaggaacaggtggtggcg). The PCR fragment obtained was digested with *FseI* and *EcoRV*. A modified version of the *aprE* promoter (P*_aprE_*) (developed and tested in [72]) was amplified by PCR from pSG-TTGACA [72] using primers PaprESG-D/*AgeI* (tgaaccggttgtcaaacatgagaattcagcg) and PaprE-R/*FseI* (caaggccggccaaattcagagtagacttacttaaaagac). The resulting PCR fragment was digested with *AgeI* and *FseI* and ligated with *FseI*/*EcoRV-*digested *dsredexpress* into *AgeI/EcoRV*-digested pLF25 in a three-point ligation to give pLFKEE (*P_spoIIE_-gfp, P_aprE_-dsred, cmp spc*). Selection of plasmid constructions in *E. coli* clones was done by adding ampicillin as described above and correct fusions were verified by sequencing.

To construct *B. subtilis* KEE, pLFKEE was transformed into *B. subtilis* 168 competent cells as previously described [58] and selected on LB solid medium supplemented with chloramphenicol. Integration clones were screened for their *amyE* phenotype on LB+1% starch solid medium [58]. The inability of the clones obtained to grow on spectinomycin was checked to eliminate single cross-over plasmid integration events. Correct integration of the fusion at the *amyE* locus was verified by PCR analysis.

### Medium-shift experimental protocol

Before each experiment, cells were streaked from −80°C glycerol stocks on LB plates with chloramphenicol and grown overnight. One colony was picked and inoculated in 5 ml liquid LB medium with chloramphenicol in a series of dilution tubes and grown overnight at 37°C. The culture the closest to OD_600_ of 1.0 was used to inoculate 60 ml of LB or GM in 250-ml flasks to a final OD_600_ of 0.05 (flask D) after elimination of the culture medium by centrifugation of the cells (6,000×g, 3 min). The culture was split in two, and successive dilutions of 1:2 were performed to a total of 5 flasks of 30 ml culture (flask D and dilution flasks: -1, -2, -3, -4). Cells in all four flasks were grown simultaneously at 37°C, 200 rpm, until the most concentrated culture grew to an OD_600_ of 1.0 (Flask D). Then, 25 ml of each culture were harvested by centrifugation (8,000×g, 5 min) and resuspended in a pre-warmed SM medium volume calculated to obtain a final OD of 0.5 (medium density). Three aliquots of 200 μl from each flask were transferred to a sterile Costar 96-well black plate with flat clear bottom (Corning). Cells in the plate were grown in a Tecan Safire microplate spectrophotometer at 37°C medium linear shaking setting (395 rpm). Culture turbidity (OD_600_) and fluorescence were measured at 15 minutes intervals for a total time of 24 hours. GFPmut2 was read at wavelengths of 481 nm (excitation) and 507 nm (emission), and DsRedexpress was read at 557 nm (excitation) and 579 nm (emission).

### Memory and mutual information analysis

There are a number of ways to translate the memory quantification definitions in Appendix S1 into an analysis algorithm. We took a simple fixed-interval, clustering-based approach executed as a five-step algorithm implemented the MATLAB© (http://www.mathworks.com/) analysis environment, as follows (see Supplemental Information Section S2 for schematic):

(step 0–select time intervals): The first step in analyzing the data is to select time intervals to analyze. We parsed the time series data (30 trajectories measured over 24 hours for each of three observables) into a ‘short-term’ set taken well before steady-state is reached (first 11 hours after t0, the onset of starvation–though we could have taken any endpoint between 8 and 19 hours and obtained the same result (see panel (b) in Section S3)) and an ‘long-term’ set. For our purposes, we take as our ‘proxy’ for long-term, asymptotic behavior the last three hours of our measurements, from 21 to 24 hours after t0, because by then all signals have remained flat for several hours. Experiments run for longer periods of time indicate that these signals remain flat for as long as we have measured them (36 hours, data not shown). However, we view this long-term data set as only a proxy for asymptotic behavior because though these signals remain constant for at least 36 hours, cells are forming spores and might be physiologically changing in other respects during this period and beyond.

(step 1–cluster data): We used the Matlab script in S2.2 to hierarchically cluster the 30 short-term and 30 long-term trajectories of each observable (10 cell histories×3 replicates) and to select ‘optimal’ clustering partitions for each. The assumption here is that the behavior of the observable (e.g., GFP intensity) falls into distinct classes, for example, increasing or decreasing. This script a) constructs a Euclidean distance matrix with the Matlab function pdist.m, b) constructs dendrograms using ward and average linkage with the function dendrogram.m, c) performs silhouette analysis on all tree cuts of both trees from (b) with the Matlab function silhouette.m [73], and d) ‘optimizes’ data clustering by selecting the partition that maximizes the mean silhouette, a measure of the compactness and separation of the clusters in the partition [73]. This step produced six 30×1 cluster vectors, one short-term and one long-term cluster vector for each of the three observables (i.e., ClustSPO_short, ClustSPO_long, ClustAprE_short, ClustAprE_long, ClustOD_short, ClustOD_short).

(step 2–estimate memory): Next we estimated the short-term and long-term memory in bits of each individual observable with the Matlab program Entropy_MutualInfo.m in S2.1. This program accepts two input vectors, A and B, and calculates from them individual informational entropies H(A) and H(B), the entropy of the pair H(A,B), and the mutual information between A and B, I(A;B) = H(A)+H(B)-H(A,B). H(X) is defined in Supplementary Information (Appendix S1), and H(X,Y) is calculated by first calculating the joint probability distribution over (X,Y) and then calculating the entropy H over this joint distribution. Thus, memory is estimated to be the mutual information between cell history and cell behavior and calculated by calling Entropy_MutualInfo.m with input vectors A = M = [1 1 1 2 2 2 …10 10 10], the cell history vector , and B equal to one of the six cluster vectors from step 1. To calculate memory fidelities, we normalized these memory estimates by dividing by H(M) = 3.32, the entropy of the cell history space.

(step 3–estimate memory in higher dimensions): The third step of the algorithm is to estimate the short and long-term memory exhibited by the combined activities of pairs of observables and by the triple of observables. To do this, we first used the script in S2.3 to combine cluster vectors from multiple read-outs. This script takes as its input two cluster vectors Clust1 and Clust2 and outputs a combined cluster vector Clust3 (e.g., if Clust1 = ClustSpo_short; and Clust2 = ClustAprE_short; then the output Clust3 is a vector capturing all combined short-term behaviors of Spo and AprE, for example (Spo,AprE) = (increasing, decreasing), (increasing, increasing) or (decreasing, decreasing)). Next, by calling Entropy_MutualInfo.m with inputs A = (the cell history vector M), and B = (the combined cluster vector Clust3), we calculate the mutual information between cell history and cell behavior, and thus the memory exhibited by the combined activity of the vector of observables contributing to Clust3. After computing short- and long-term memory for all four possible vector combinations of the observables, these estimates were divided by H(M) to estimate memory fidelities and normalized according to Definition (6) in Methods to estimate memory orthogonalities. Finally, we (step 4) calculated the mutual information between all pairs of observables using the cluster vectors from (step 1) as inputs to Entropy_MutualInfo.m.

We took this fixed-interval, clustering-based approach because of our desire to focus on how different cell histories can lead to qualitatively different stress response behaviors, and because a much larger data set would be required to use algorithms such as that suggested by Swinney to estimate mutual information at measurement intervals short enough to avoid excessive ‘blurring’ of the time series dynamics [74], [75]. See Section S3 in Supplemental Information for a detailed discussion of alternative approaches and why we chose the one we did, and Section S2 for Matlab scripts and programs, including a note on a bootstrap method for calculating confidence intervals that one could apply to data sets with a sufficient number of replicates (not present in this data set).

## Supporting Information

Appendix S1 Memory quantification definitions.
(0.19 MB PDF)

Section S1 Fundamental limitations of memory experiments.
(0.27 MB PDF)

Section S2 Matlab programs and flow chart for memory analysis.
(0.27 MB PDF)

Section S3 Alternative strategies for memory calculations.
(0.16 MB PDF)

Table S1 Complete set of memory and mutual information calculations.
(0.72 MB PDF)

Table S2 Bacillus strain and plasmid table.
(0.12 MB PDF)

Figure S1 Example histograms from flow cytometry analysis of *B. subtilis* strain KEE.
(0.84 MB PDF)

Figure S2 Sporulation initiation delay as a function of cell history.
(0.13 MB PDF)

Figure S3 The P*spoIIE-gfp* fusion activity tracks *spoIIE* gene expression.
(0.25 MB PDF)
