# Supplementary material for: Memory in Microbes: Quantifying History-Dependent Behavior in a Bacterium
Source: PLoS One. 2008 Feb 27;3(2):e1700. doi: 10.1371/journal.pone.0001700 (PMC2264733; doi:10.1371/journal.pone.0001700)
Supplement: Appendix S1 — Memory quantification definitions. (0.19 MB PDF) [file pone.0001700.s001.pdf]

Supplementary Information S1

For "Memory in Microbes: Quantifying History-Dependent Behavior in a Bacterium", by Denise M. Wolf, Lisa Fontaine-Bodin, Ilka Bischofs, Gavin Price, Jay Keasling, and Adam P Arkin. PLoS ONE 2008

Appendix S1. Memory quantification definitions

1. ***(Adaptive) memory experiment compendium:*** A set of experiments in which populations of one or more cells are subjected to distinct treatments *m* prior to time t0, and an identical treatment *S* following t0, with at least one observable read-out of behavior *Y* sampled over a time series following t0. The set of all data collected from the memory experiment compendium, viewed as a measurement-transformed sampling of the map from past cell history to future cellular response, is called the *memory data compendium*.

2. ***Entropy of cell history space tested:*** Within the memory experiment compendium, cell history *M* is considered a random variable. If M is the set of all experimentally tested cell treatments/histories *m* prior to t0 that *M* could be, and $p(m)$ = $\Pr(M = m)$, then M has $H(M) = -\sum_{m \in \mathrm{M}} p(m)\log_2(p(m))$ bits of informational entropy [1].

3. ***Long term memory:*** Let t_{asym} be the time it takes for the observable response *Y* to approach steady state. Then within M, the cells under study exhibit $\mathrm{I}_{\mathrm{asym}}(M;Y) \equiv \mathrm{I}(M;Y(t=t0+t_{asym}:\infty))$ bits of *long term memory* in the observable response *Y* to stress condition *S*.

4. ***Short term memory:*** Let $t_{trans} < t_{asym}$. Though short-term, transient behavior (and memory) may be measured over any interval or at any time point between t0 and t_{asym} (see the discussion in SI.2) we use the interval [t0 t_{trans}] in our memory calculations as follows: within M the cells under study exhibit $\mathrm{I}_{\mathrm{trans}}(M;Y;\, t_{trans}) \equiv \mathrm{I}(M;Y(t = t0:t0+ t_{trans}))$ bits of *short term memory* over (t0: t0+ t_{trans}) in the observable response *Y* to stress condition *S*.

5. ***Memory fidelity:*** The *short-term memory fidelity* exhibited over (t0: t0+ t_{trans}) in response Y of the cells to stressor *S* given M is $\mathrm{P}_{\mathrm{trans}}(M;\ Y;\ t_{trans}) \equiv \mathrm{I}(M;Y(t = t0:t0+t_{trans}))/\mathrm{H}(M)$. The *long-term memory fidelity* exhibited in response Y of the cells to stressor *S* given the cell history space M is $\mathrm{P}_{\mathrm{asym}}(M;Y) \equiv \mathrm{I}(M;Y(t = t0+ t_{asym}:\infty))/\mathrm{H}(M)$. This normalized mutual information metric, a measure between 0 and 1 of the fraction of uncertainty about the past conditions tested that is reduced by knowledge of future cellular response, has also been called the coefficient of constraint [2].

6. ***Memory orthogonality***: The *memory orthogonality* between two pathway responses *Y1* and *Y2* of cells subjected to stress condition *S* given cell histories M is: $Mem_{orth}(M;(Y1,Y2)) \equiv (I(M;(Y1,Y2))-max(I(M;Y1),I(M;Y2)))/min(I(M;Y1),I(M;Y2))$. Mem_{orth} equals 1 if the two variables combined as a vector yield the upper bound of memory under these conditions, and 0 if the two variables in combination yield the lower bound (a consequence of the inequality

$min(I(M;Y1),I(M;Y2)) \leq I(M;(Y1,Y2)) \leq I(M;Y1)+ I(M;Y2)$ [2]). This definition extends naturally to output triples and higher order combinations, as well.

References

1. Shannon CE (1948) A mathematical theory of cummunication. Bell Systems Technical Journal 27: 379–423 and 623–656.

2. Ash RB (1990) Information Theory: Dover Publications.
