# Supplementary material for: Memory in Microbes: Quantifying History-Dependent Behavior in a Bacterium
Source: PLoS One. 2008 Feb 27;3(2):e1700. doi: 10.1371/journal.pone.0001700 (PMC2264733; doi:10.1371/journal.pone.0001700)
Supplement: Section S1 — Fundamental limitations of memory experiments. (0.27 MB PDF) [file pone.0001700.s002.pdf]

Supplementary Information Section S1

For "Memory in Microbes: Quantifying History-Dependent Behavior in a Bacterium", by Denise M. Wolf, Lisa Fontaine-Bodin, Ilka Bischofs, Gavin Price, Jay Keasling, and Adam P Arkin. PLoS ONE 2008

S1. Fundamental limitations of memory experiments

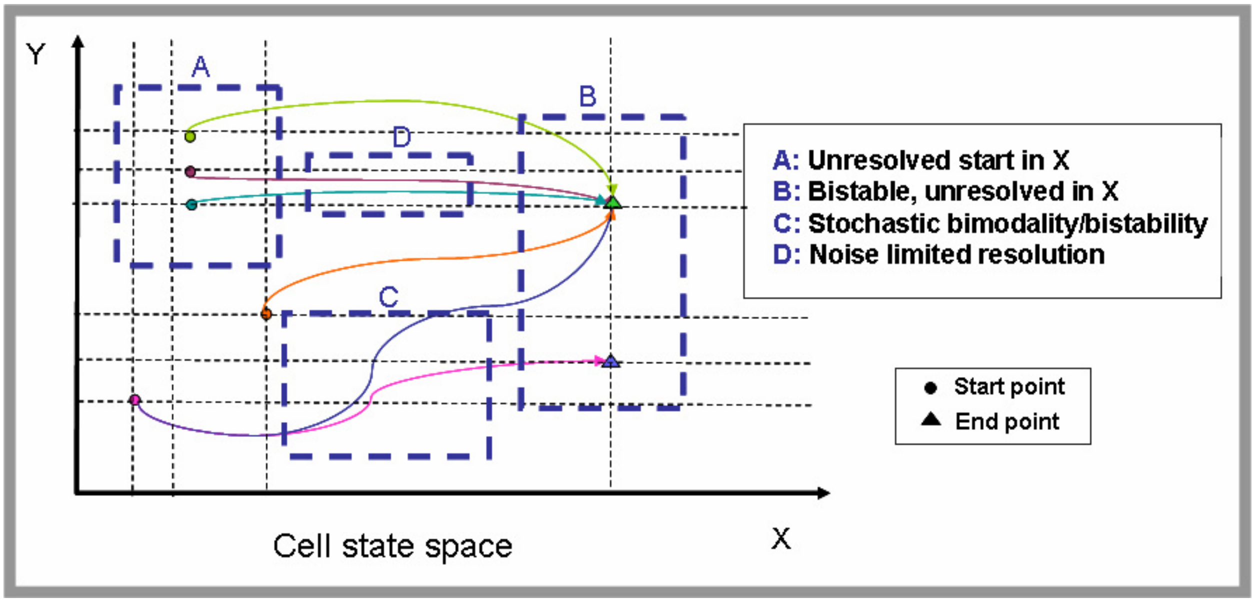

Figure. Any attempt to quantify or classify memory is fundamentally limited by the possibility of unobservable states, uncontrolled and unobservable inputs, and measurement errors and distortions. This figure shows a variety of initial conditions (start points, circles) in a toy cell state space, each corresponding to a different cell history up to time t0. After t0, the system evolves over time to representative asymptotic behaviors (end points, triangles), including a deterministic steady state (green triangle in box B), bistability (both triangles in box B), and stochastic bistability (boxes C,B). Box A shows distinct initial conditions that are observable if both X and Y are observed or only Y is observed, but not if only X is observed. Thus, the amount of history-dependence in the system would be underestimated if only X were observed. Likewise, box B shows distinct asymptotic states that are not observable if only X is measured. The three trajectories starting in box A also demonstrate how memory of initial conditions can decrease over time, possibly even with no long term memory at all. The trajectories passing through box D are resolvable only until measurement noise overwhelms the signal. If measurements are averaged over the population, stochastic or multimodal features of the trajectory (boxes C, B) become invisible, whereas if measurements are taken over the population in the form of histograms (flow cytometry), a population consisting of cells that stochastically alternate between 'off' and 'on' states is indistinguishable from a population of cells that switch into one of the two states and then remain in that state for the duration of the experiment..

Unobservable states: In a perfect world, one would take direct, noiseless, high-resolution measurements of every state variable in the cell and its environment, and exercise perfect control over input perturbations. Real world experiments suffer from limitations due to imperfect observation and input control, lack of knowledge of the space of meaningful biological inputs,

and finite time and resources. These limitations necessarily constrain our ability to detect, classify, and quantify memory. One major limitation derives from the presence of unobserved cell state variables A real-world observer selects a small subset of state variables to observe and measure, thereby collapsing the high-dimensional state space of a cell or population into a low-dimensional projection. Collapsed dynamics can lead to misclassifying and misquantifying memory.

Though physical first principles predict that chemistry within cells should behave like Markov processes, the presence of unobserved states can make a Markov memory appear to be non-Markov. If, for example, two initial conditions differ only in the y axis but have the same value along the x axis (Figure, box A), an observation of only the x-component of the two diverging trajectories would suggest to a naïve observer that the system behaviour depends not only on the initial condition, but also on the path leading to that initial condition. For similar reasons, unobserved states can also lead to underestimates of transient and asymptotic memory. Two system trajectories responding to two distinct cell histories may appear to be identical from the perspective of the low-dimensional observations, yet be perfectly distinct in the higher-dimensional native state space (Figure, boxes A,B).

Uncontrolled, unobserved system inputs can also lead to misclassifying and misquantifying memory, though the errors are different. If one were to repeat an experiment where a stimulus is applied and the system response is measured, and there are uncontrolled, unobserved, randomly varying input components that the observer is not aware of, a deterministic system could be misclassified as stochastic. If the uncontrolled, unobserved input correlates with the controlled portion of the input stimulus, one could overestimate the ability of the system to 'remember' the specific observed stimulus, though not the overall memory of the system.

Measurement distortions, information loss and errors: The next layer of challenge in quantifying cellular memory derives from imperfect and distorting measurement of cellular response. Distortions and information loss arise from proxy measurements, instrument error, experimental error, time discretization/integration, and from indirect measurement modalities. Fluorescent transcriptional fusions that supposedly measure promoter activity introduce their own protein transcription, translation, folding, and degradation dynamics, and can cause toxicity and therefore perturb the system being measured. Errors from proxy measurements combine with those from instrument error, experimental error, and information loss from time discretization/integration to limit the resolution and dynamic range of the behavior we can observe. This in turn can lead to underestimates of the amount of memory in a system (quantification errors). The cell may have distinct trajectories and attractor states associated with distinct cell histories, but if the trajectories and states cannot be resolved due to the 'noise' in these various types of measurement error (Figure, box D), an underestimation of the memory capacity of the system will result. Classification errors can result too, for example a stochastic response that appears deterministic because the distribution lies within measurement noise bounds.

Measurement modalities operating on bulk, averaged populations are especially prone to classification ambiguities. With bulk measurements as one obtains with fluorimetry, for example, averaged measurements over populations of cells conflate deterministic behavior (all

cells express some GFP) and stochastic bistability (some cells express a high level of GFP and others express none) (Figure, boxes B, C). This can lead to a memory classification error if one assumes the average measurement reflects individual cell behavior. A memory quantification error (underestimate) can also result, if the average response to distinct cell histories is the same but the single-cell response distributions differ.

Population distribution measurements as one obtains through flow cytometry are less prone to classification errors, as they can distinguish between deterministic behavior and stochastic behavior, but they still suffer ambiguities discriminating between stochastic bistability associated with rapid transitioning between states bistability with little if any transitioning between states on experimental time scales. They also lose information due to the limited dynamic range of the machine, finite binning of fluorescence levels, different sized cells, differential cell growth and death rates among cell subpopulations in different states, and the inability to follow single cell fates. Even single-cell measurements from time-lapse microscopy suffer from sampling errors, image processing errors, errors due to fluctuating illumination and drift, and difficulty identifying rare events. All of these errors distort trajectories and can lead to underestimations of memory.

Because these limitations are inherent in the estimation of memory processes and most likely result in underestimates of the ability of the system to ‘remember’ the cell histories tested by the experimental compendium, we interpret quantifications of memory within our compendium as lower bound estimates.
