# Supplementary material for: Memory in Microbes: Quantifying History-Dependent Behavior in a Bacterium
Source: PLoS One. 2008 Feb 27;3(2):e1700. doi: 10.1371/journal.pone.0001700 (PMC2264733; doi:10.1371/journal.pone.0001700)
Supplement: Section S2 — Matlab programs and flow chart for memory analysis. (0.27 MB PDF) [file pone.0001700.s003.pdf]

Supplementary Information Section S2

For "Memory in Microbes: Quantifying History-Dependent Behavior in a Bacterium", by Denise M. Wolf, Lisa Fontaine-Bodin, Ilka Bischofs, Gavin Price, Jay Keasling, and Adam P Arkin. PLoS ONE 2008

S2. Matlab codes for memory analysis

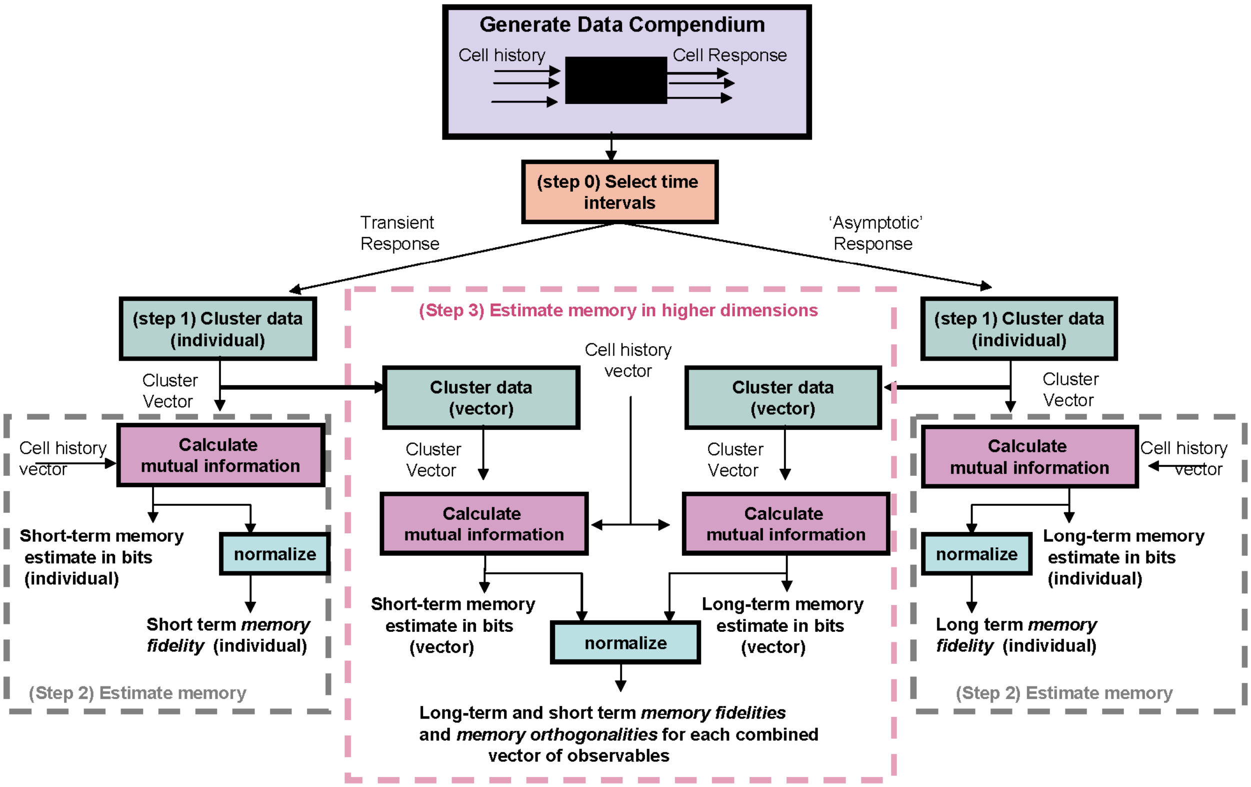

Flow chart. Schematic of a fixed-interval, information-based approach to quantifying memory in bacteria (A3 in the figure in Section S3). As described in Materials and Methods, in our implementation of a fixed-interval approach to memory quantification in bacteria we first (step 0) parse the time series data into a transient set and a long-term 'asymptotic set'. Then, for the transient and asymptotic data sets, respectively, we: (step 1) use the Matlab scripts in S2.2 to hierarchically cluster the trajectories and select an 'optimal' partition using the Silhouette criterion, a clustering validation and optimization technique based on maximizing the compactness and separation of the clusters in a partition [1]. This step produced six cluster vectors, one transient and one 'asymptotic' cluster vector for each of the three observables (i.e., ClustSPO_trans, ClustSPO_asym, ClustAprE_trans, ClustAprE_asym, ClustOD_trans, ClustOD_asym); (step 2) calculate memory in bits as the mutual information between cell history and cell behavior cluster for each observable on each time-scale using the Matlab program in S2.1 with input vectors ClustA=M=[1 1 1 2 2 2 …10 10 10] as the cell history vector and ClustB equal to one of the six cluster vectors from step 1. To calculate memory fidelities, we normalized the memory estimates by dividing by H(M)=3.32, the entropy of the cell history space; (step 3) calculate memory in bits exhibited by pairs of observables and by the triple of observables by (a) using the script in S2.3 to combine cluster vectors from multiple read-outs (e.g., Clust1=GFP cluster vector; Clust2=DsRed cluster vector; Clust3=combined (GFP,DsRed) cluster vector) and (b) using as inputs to the program in S2.1, ClustA=(the cell history vector M) and ClustB=(the combined cluster vector Clust3), calculate the mutual

information between cell history and cell behavior in the higher dimensional spaces. These estimates are normalized by H(M) to estimate *memory fidelities* and by Equation (1) to estimate *memory orthogonalities*. Finally, we (step 4, not shown) calculate the mutual information between the observables using the program in S2.1. Though we did not have enough replicates to meaningfully do so in this paper, with enough replicates one could estimate confidence intervals for all memory and mutual information bit counts by performing a statistical (non-parametric) bootstrap analysis. Such an analysis would involve random sampling with replacement from the replicate sets, followed by clustering and mutual information calculations for each derived data set to generate a distribution of memory estimates. From this distribution, confidence intervals could easily be determined.

S2.1) *Matlab program to calculate memory and mutual information (Steps 2-4 in the data analysis algorithm in Materials and Methods).*

```
%%%%%%%%%%%%%%%%%%%%%%%%%%%%%%%%%%%%%%%%%%%%%%%%%%%%%%%%%%%%%%%%%%%%%%
%Function: Entropy_MutualInfo.m
%
%Description: This MATLAB program accepts two input vectors, A and B,
%and calculates from them individual entropies H(A) and H(B),  the
%entropy of the pair H(A,B), and the mutual information between A and
%B: I(A;B) = H(A)+H(B)-H(A,B).
%
%Inputs:
%     ClustA='optimal' clustering vector for A (each entry an integer)
%     ClustB='optimal' clustering vector for B (each entry an integer)
%Outputs:
%     HA=entropy of A in bits
%     HB=entropy of B in bits
%     HAB=entropy of the vector (A,B)in bits
%     IAB=mutual information of A and B in bits
%
%Interpretation: IF (A=cell history) and (B=Response)
%                THEN memory = IAB. IF (A=response 1) and (B=Response2)
%                THEN mutual information = IAB and memory in combined
%                     response vector = HAB (assuming cell history
%                     space uniformly distributed).
%
%Author:  Denise Wolf dmwolf@lbl.gov
%%%%%%%%%%%%%%%%%%%%%%%%%%%%%%%%%%%%%%%%%%%%%%%%%%%%%%%%%%%%%%%%%%%%%%
%%%%%%%%%%%%%%%%%%%%%%%%%%%%%%%%%%%%%%%%%%%%%%%%%%%%%%%%%%%%%%%%%%%%%%

function  [HA,HB,HAB,IAB]=Entropy_MutualInfo(ClustA,ClustB)


no_clustersA=max(ClustA);
no_clustersB=max(ClustB);


%H=-sum(log2(pi)pi)  = informational entropy

HA=0;
```

```
for i=1:no_clustersA
  size_clustiA(i)=length(find(ClustA==i));
  piA(i)= size_clustiA(i)/length(ClustA);
  HA=HA-log2(piA(i))*piA(i);
end

HB=0;

for i=1:no_clustersB
  size_clustiB(i)=length(find(ClustB==i));
  piB(i)= size_clustiB(i)/length(ClustB);
  HB=HB-log2(piB(i))*piB(i);
end


%%%I(X,Y)=H(X)+H(Y)-H(X,Y)= mutual information of X,Y
%%H(X,Y)=sum_x(sum_y(pij*log2(pij))= entropy of vector (X,Y)

PAB=zeros(no_clustersA,no_clustersB); %PAB is the probability distribution of
A,B
HAB=0;
for j=1:no_clustersB
    for i=1:no_clustersA

PAB(i,j)=length(intersect(find((ClustA==i)),find((ClustB==j))))/length(ClustA
);
        if PAB(i,j)>0
           HAB=HAB-PAB(i,j)*log2(PAB(i,j));
        end
    end
end


IAB=HA+HB-HAB;

%%%%%%%%%%%%%%%%%%%%%%%%%%%%%%%%%%%%%%%%%%%%%%%%%%%%%%%%%%%%%%%%%%
%%%%%%%%%%%%%%%%%%%%%%%%%%%%%%%%%%%%%%%%%%%%%%%%%%%%%%%%%%%%%%%%%%
```

S2.2) *Matlab scripts for visualizing and clustering data (Step 1).*

```
%Input:   Data = m x n time series matrix, where m = the number of
%         trajectories and n= the number of time points.
%         History_Labels = m x 1 text vector with cell history labels.
%
% Directions: Run this script twice, first with the ward linkage and
%    second with the average linkage, and select the partition
%    (Tx; x∈[1,7]) that maximizes the mean silhouette.  We run this script
%     twice because the two linkage functions can produce
%     different trees, and thus different partitions. The ‘best’ partition of
%     the 14 calculated, according the silhouette criterion
%     (maximum mean silhouette over all clusters, for seven cutoffs each from
%     the trees constructed using average and ward linkages) is then selected
```

```
%     as the 'optimal' clustering output of the procedure.
%%%%%%%%%%%%%%%%%%%%%%%%%%%%%%%%%%%%%%%%%%%%%%%%%%%%%%%%%%%%%%%%%%%%%%%%%%%%%%%%%%%

%Calculate distance matrix
Data_dist=pdist(Data,'euclidean');

%Calculate linkage function
Zfunction_ward=linkage(Data_dist,'ward');
Zfunction_avg=linkage(Data_dist, 'average');
Zfunction=Zfunction_ward;  %or avg

%Visualize clustering over all cell histories
figure;[H,T,perm] = dendrogram(Zfunction,0, 'colorthreshold','default',
'orientation','left','labels',History_Labels,'colorthreshold',4.5);

figure   %Calculate cluster vectors T for the first 7 levels of tree-cut
[H,T2,perm] = dendrogram(Zfunction,2, 'colorthreshold','default',
'orientation','left','labels',History_Labels);
[H,T3,perm] = dendrogram(Zfunction,3, 'colorthreshold','default',
'orientation','left','labels', History_Labels);
[H,T4,perm] = dendrogram(Zfunction,4, 'colorthreshold','default',
'orientation','left','labels', History_Labels);
[H,T5,perm] = dendrogram(Zfunction,5, 'colorthreshold','default',
'orientation','left','labels', History_Labels);
[H,T6,perm] = dendrogram(Zfunction,6, 'colorthreshold','default',
'orientation','left','labels', History_Labels);
[H,T7,perm] = dendrogram(Zfunction,7, 'colorthreshold','default',
'orientation','left','labels', History_Labels);

figure;
subplot(3,3,1)  %3x3 plot, first figure= visualize 2-d projection
[Y,eigvals] = cmdscale(Dfunction);
plot(Y(:,1),Y(:,2),'.','MarkerSize',15);
text(Y(:,1),Y(:,2),History_Labels,'FontSize',7);
xlabel('MDS scaled distance')
ylabel('MDS scaled distance')

subplot(3,3,2)   %Second figure = silhouettes for 2-level tree cut
[s2,h2]= silhouette(Data,T2,'euclid');

subplot(3,3,3)   %Third figure = silhouettes for 3-level tree cut
[s3,h3]=silhouette(Data,T3,'euclid');

subplot(3,3,4)    %Fourth figure = silhouettes for 4-level tree cut
[s4,h4]=silhouette(Data,T4,'euclid');

subplot(3,3,5)    %Fifth figure = silhouettes for 5-level tree cut
[s5,h5]=silhouette(Data,T5,'euclid');

subplot(3,3,6)    %Sixth figure = silhouettes for 6-level tree cut
[s6,h6]=silhouette(Data,T6,'euclid');

subplot(3,3,7)    %Seventh figure = silhouettes for 7-level tree cut
 [s7,h7]=silhouette(Data,T7,'euclid');
```

```
subplot(3,3,8)    %Eighth figure = mean silhouette for each partition
bar([2:1:7],[mean(s2) mean(s3) mean(s4) mean(s5) mean(s6) mean(s7)])
xlabel('Number of clusters in partition')
ylabel('Mean Silhouette')

end
```

Caveat: Silhouette is a popular cluster validation/selection measure that scores a partition based on both the compactness of its clusters and their separation [1]. People like it because it is systematic, and because it takes into account local and global properties of a partition. However, like all clustering validation/selection measures, Silhouette has its biases. It is a conservative criterion in that there is a tendency for the method to swamp out detail and under-report the number of clusters if there are multiple widely-spaced scales of cluster separation.

For the data set analyzed in this paper, this is most evident in the clustering of the transient data set for growth (OD_{600}). By eye it appears that there might be some structure in the data set at early time points (OD_{600} of cells with a history of growth in GM, but to different densities, figs. 5c and 6c) that gets subsumed into two large clusters because of the large separation between the set faster growing trajectories with a history of growth in GM and the much slower growing set of trajectories with a history of growth in LB. Despite this conservatism, we used Silhouette because of all the methods we considered, it seemed to do the best job selecting tree cuts (mostly because the majority of our data is structured on a single level of resolution). However, given the difficulty in selecting a good clustering validation technique, we suggest that the reader take the structure of their data sets into account when selecting a clustering criterion so as to get the best 'match' between the bias of the method and the data to be analyzed.

S2.3) *Matlab script for combinatorially combining cluster vectors (Step 2-3).*

```
%%%%%%%%%%%%%%%%%%%%%%%%%%%%%%%%%%%%%%%%%%%%%%%%%%%%%%%%%%%%%%%%%%%%%%%%%%%%
%Input:   Clust1  - the cluster vector from one observable (integers)
%         Clust2  - the cluster vector from another observable (integers)
%
%Output:  Clust3 – the cluster vector obtained by combinatorially combining
%           the cluster vectors ClustA and ClustB.
%
%Example:  Say Clust1 = [ 1 1 2 2 2] and Clust2 = [ 1 1 1 2 2]. Then Clust3
%          =[1 1 2 3 3] to show that there are three possible combinations:
%      (Clust1,Clust2)=(1,1); (Clust1,Clust2)=(2,1) and (Clust1,Clust2)=(2,2)
%%%%%%%%%%%%%%%%%%%%%%%%%%%%%%%%%%%%%%%%%%%%%%%%%%%%%%%%%%%%%%%%%%%%%%%%%%%%

  D=[Clust1 Clust2];
  Clust3=clusterdata(D,0.1);    %clusterdata.m is a function in the Statistics
                          %                 Toolbox of Matlab.
```

Reference

1. Rousseeuw PJ (1987) Silhouettes: A graphical aid to the interpretation and validation of cluster analysis. Journal of Computational and Applied Mathematics 20: 53-65.
