# Supplementary material for: Memory in Microbes: Quantifying History-Dependent Behavior in a Bacterium
Source: PLoS One. 2008 Feb 27;3(2):e1700. doi: 10.1371/journal.pone.0001700 (PMC2264733; doi:10.1371/journal.pone.0001700)
Supplement: Section S3 — Alternative strategies for memory calculations. (0.16 MB PDF) [file pone.0001700.s004.pdf]

Supplementary Information Section S3

For "Memory in Microbes: Quantifying History-Dependent Behavior in a Bacterium", by Denise M. Wolf, Lisa Fontaine-Bodin, Ilka Bischofs, Gavin Price, Jay Keasling, and Adam P Arkin. PLoS ONE 2008

S3. Alternative strategies in time-series interval sampling for memory calculations:

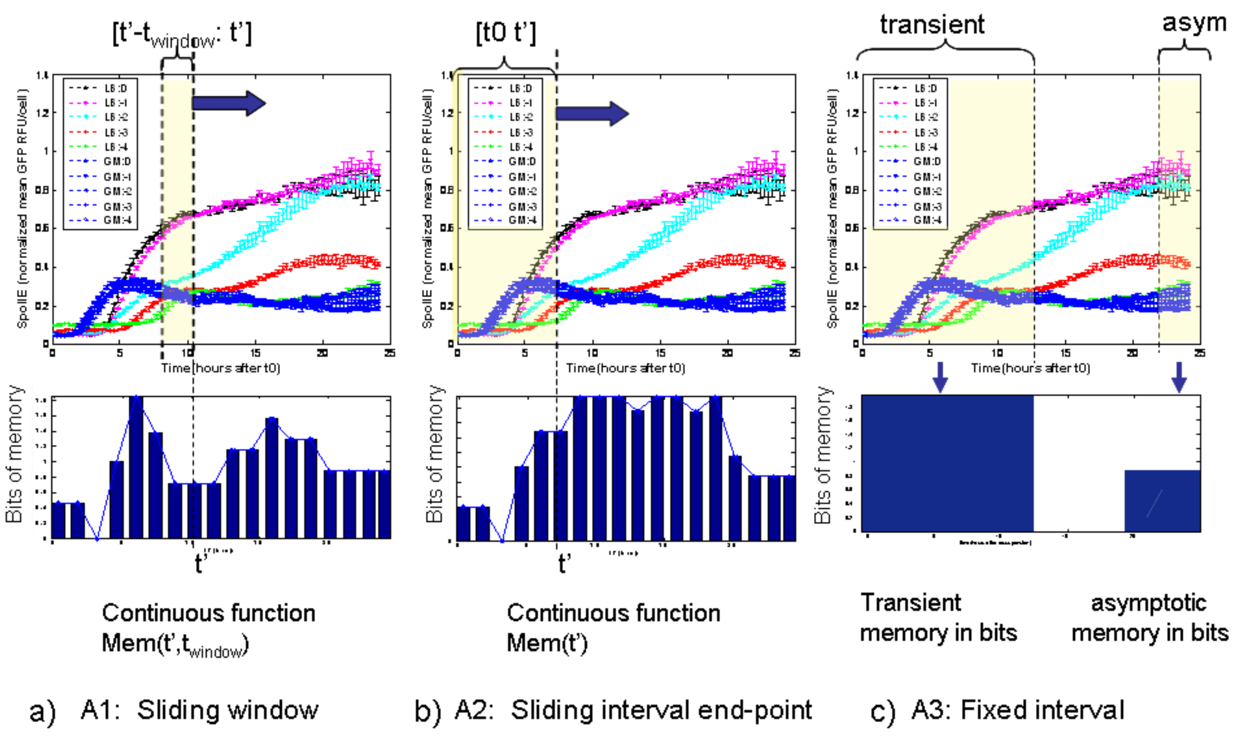

Figure. Three possible approaches to interval sampling for memory calculations: a) sliding window (A1), b) sliding interval end-point (A2), and c) fixed interval (A3).

Memory calculations for adaptive memory experiments involve calculating the mutual information between cell history before time t0 and cell behavior after time t0. If cellular behavior is measured at a single time point, say at 24 hours after t0, it would be clear how to proceed, at least in terms of temporal sampling of the response: one would calculate the mutual information between the cellular response measurements at that single time point and the cell histories corresponding to each measurement. However, because in our experiments behavior after time t0 is measured as a time series – every 15 minutes for 24 hours – a choice must be made about which time points or intervals to analyze.

On one end of the spectrum is a sliding window approach (which we will call A1). If the window size is 1, admitting just a single time point, this approach amounts to calculating the mutual information between cell history and cell response at each sampled time point after t0. Or one could select a window size greater than 1, and 'slide' the window along the time axis, producing a calculation for mutual information between cell history and response from $t'-t_{window}$ to t', as t' ranges from $t0+t_{window}$ to the final measurement at t0+24 hours. The sliding window approach produces a (sampled) continuous time-varying memory measurement function, as shown in panel (a) of the above figure for a window size of 5 sample points (≈1.5 hours) applied to the sporulation initiation time series data. (Though Swinney's information analysis algorithm is in general appropriate for a sliding window approach, we used the clustering and silhouette analysis approach described in Supplementary Information Section S2 for this example because a much larger data set than ours would be required to use Swinney's analysis algorithm at measurement intervals short enough to avoid excessive 'blurring' of the time series dynamics [1] [2]).

On the other end of the spectrum is a fixed-interval approach (approach A3). This approach involves extracting transient and asymptotic segments of the temporal cellular response. The mutual information between the transient response and cell history is then used to estimate transient memory, whereas the mutual information between the asymptotic response and cell history is used to estimate asymptotic memory. This approach is discrete, in that it maps a time series onto just two numbers – the transient and asymptotic bit counts, as shown in panel (c) of the above figure for the sporulation initiation time series data.

Between these two ends of the spectrum is the sliding interval end-point approach (approach A2). In this approach all analyzed measurement intervals start at time t0, but the end point is variable, or sliding, starting from t0 and ending at the final time point, t0+24hr. Panel (b) in the above figure shows the result of this approach applied to the sporulation initiation time series data.

Each of the three approaches has its pros and cons. Both the sliding window (A1) and the sliding interval end-point (A2) approaches are appealing in that the measures are continuous, and as the final time gets very large relative to time it takes to converge to asymptotic behavior, both measurements converge to the asymptotic value. Another interesting aspect of these approaches is that they communicate the temporal increase and decrease of information and mutual information from the perspective of the observer. The increase captures, among other (conflated) dynamics, the rate at which the history-dependent states of the cells become observable in the read-out, whereas the decrease captures (also among other conflated dynamics) the decay rate of memory as it relaxes from the maximum amount of transient memory to the lesser long-term memory. This notion of memory decay time could also be mapped onto a single measurement like a half-life - the time after t0 after which the amount of memory remains at less than half of the difference between the maximum transient memory and the long term memory. One potential down-side, however, especially for the sliding window method, is the emphasis on the moment experience of an outside observer of as increases and decreases in information and memory due to 'curve crossing' resulting from unobservable states are traced (see panel (a) in the above figure). This emphasis is a departure from our goal of quantifying alternative pathway control

conditioned by cell history. A naive interpretation of memory curves derived from these approaches could also be misleading. For instance, looking at panels (a-b) in the figure, one might conclude that the cells mostly do not 'remember' their history for the first few hours, and then start to remember their history when the curves diverge. However, since cell history prior to t0 is most likely to impact cell behavior after t0 largely through an initial condition of the cell as a whole at time t0, a more likely interpretation is that though the cells encode memory of their past experiences maximally at time t0, the manifestation of this memory in the observable does not become apparent for several hours.

Consequently, given our interest in capturing substantial qualitative differences in transient and long-term patterns of behavior of the pathways as conditioned on prior history, we chose the discrete, fixed-interval approach (A3). Though this approach is not as visually interesting as the continuous information and memory curves derived from A1 and A2, we believe it is more consistent with our goals of calling out history-dependent behavior patterns and is appealing in its relative simplicity.

One issue with the fixed-interval method we chose is that one must choose time intervals to analyze. This may be done ad hoc, according to the interests of the analyst, or more systematically. One way to approach interval selection systematically is to perform a sliding interval end-point analysis, as shown in (b) above for the sporulation time series, to determine the information dynamics of the curves as a function of interval. If the goal is to pick a transient interval that maximizes information & memory, the end point can be selected by choosing a time point at the maximum on the curve. For our data, intervals that start at t0 and end anywhere from 8 to 19 hours after t0 are roughly equivalent, informationally speaking. We selected 11 hours after t0 to be the end point of the transient data interval, but would have obtained the same results had we chosen nearly any point between 8 and 19 hours after t0.

References

1. Vastano JA, Swinney HL (1988) Information transport in spatiotemporal systems. Physical Review Letters 60: 1773-1776.
2. Samoilov M, Arkin A, Ross J (2001) On the deduction of chemical reaction pathways from measurements of time series of concentrations. Chaos 11: 108-114.
