# Supplementary material for: Memory in Microbes: Quantifying History-Dependent Behavior in a Bacterium
Source: PLoS One. 2008 Feb 27;3(2):e1700. doi: 10.1371/journal.pone.0001700 (PMC2264733; doi:10.1371/journal.pone.0001700)
Supplement: Table S1 — Complete set of memory and mutual information calculations. (0.72 MB PDF) [file pone.0001700.s005.pdf]

Mutual information across observable output pairs in bits

Memory of cell history in bits

	AprE asym	SpoIIE asym	OD_{600} asym	AprE trans	SpoIIE trans	OD_{600} trans
**AprE asym**	**0.7219**	0.4464	0.3219	0.7219	0.7219	0.2365
**SpoIIE asym**	1.1568	**0.8813**	0.5568	0.6058	0.8813	0.3958
**OD_{600} asym**	1.371	1.2955	**0.971**	0.6955	0.971	0.61
**AprE trans**	1.4855	1.761	1.761	**1.4855**	1.4855	1
**SpoIIE trans**	1.961	1.961	1.961	1.961	**1.961**	1
**OD_{600} trans**	1.4855	1.4855	1.361	1.4855	1.961	**1**

Table S1: Complete set of memory and mutual information calculations. Estimated lower bounds on cell-history memory (red, lower left triangle) and mutual information (blue, upper right triangle) in informational entropy bits for each individual *B. subtilis* stress response read-out and each vector pair of read-outs, with respect to the cell histories tested by our compendium. Read-outs include both transient and 'asymptotic' sporulation initiation (P*spoIIE-gfp* expression), AprE synthesis (P*aprE-dsred* expression), and growth (OD_{600}) signals. For example, the mutual information between transient sporulation and AprE signals can be found in the 4th row and 5th column of the matrix (1.4855 bits) whereas the memory encoded in the combined transient sporulation and AprE signals can be found in the 5th row and 4th column of the matrix (1.961 bits). Notice that the upper right 3x3 off-diagonal block contains estimates of the mutual information between all combinations of transient and long-term signals, which provides an estimate for how much transient memory carries over into the long term response. See Materials and Methods for details on the calculations.
