# Supplementary material for: Memory in Microbes: Quantifying History-Dependent Behavior in a Bacterium
Source: PLoS One. 2008 Feb 27;3(2):e1700. doi: 10.1371/journal.pone.0001700 (PMC2264733; doi:10.1371/journal.pone.0001700)
Supplement: Table S2 — Bacillus strain and plasmid table. (0.12 MB PDF) [file pone.0001700.s006.pdf]

Strains and plasmids	Genotype	Reference
Bacillus subtilis		
168	*trpC2*	[1]
LF25	168, *amyE*::P_{spoIIE}-*gfp*, *cmp*	This study
KEE	168, *amyE*::P_{spoIIE}-*gfp*, P_{aprE}-*dsred*, *cmp*	This study
**Plasmids**		
pMF19	P_{spoIIG}–*gfp*, *spc*	[2]
pEA18	P_{xyl}-*gfp*, *cmp spc*	[3]
pLF22	P_{spoIIG}–*gfp*, *cmp spc*	This study
pLF25	P_{spoIIE}-*gfp*, *cmp spc*	This study
pSG-TTGACA	$P_{aprE'}$-*lacZ*, *cmp*	[4]
pDsRed-Express	P_{lac}-*DsRed-Express*, *amp*	Clontech
pLFKEE	P_{spoIIE}-*gfp*, P_{aprE}-*dsred*, *cmp spc*	This study

Table S2. Bacillus strains and plasmids table. The experiments in this paper made use of these bacterial strains and plasmids.

References

1. Kunst F, Ogasawara N, Moszer I, Albertini AM, Alloni G, et al. (1997) The complete genome sequence of the gram-positive bacterium Bacillus subtilis. Nature 390: 249-256.
2. Fujita M, Losick R (2002) An investigation into the compartmentalization of the sporulation transcription factor sigmaE in Bacillus subtilis. Mol Microbiol 43: 27-38.
3. Quisel JD, Lin DC, Grossman AD (1999) Control of development by altered localization of a transcription factor in B. subtilis. Mol Cell 4: 665-672.
4. Jan J, Valle F, Bolivar F, Merino E (2001) Construction of protein overproducer strains in Bacillus subtilis by an integrative approach. Appl Microbiol Biotechnol 55: 69-75.
