# Supplementary material for: Memory in Microbes: Quantifying History-Dependent Behavior in a Bacterium
Source: PLoS One. 2008 Feb 27;3(2):e1700. doi: 10.1371/journal.pone.0001700 (PMC2264733; doi:10.1371/journal.pone.0001700)
Supplement: Figure S1 — Example histograms from flow cytometry analysis of B. subtilis strain KEE. (0.84 MB PDF) [file pone.0001700.s007.pdf]

S1. The distribution over the population of GFP and DsRedExpress expression levels for selected cell histories and time points.

The histograms below derive from a flow cytometric analysis of the *B. subtilis* reporter strain KEE at selected time points and for selected cell histories.

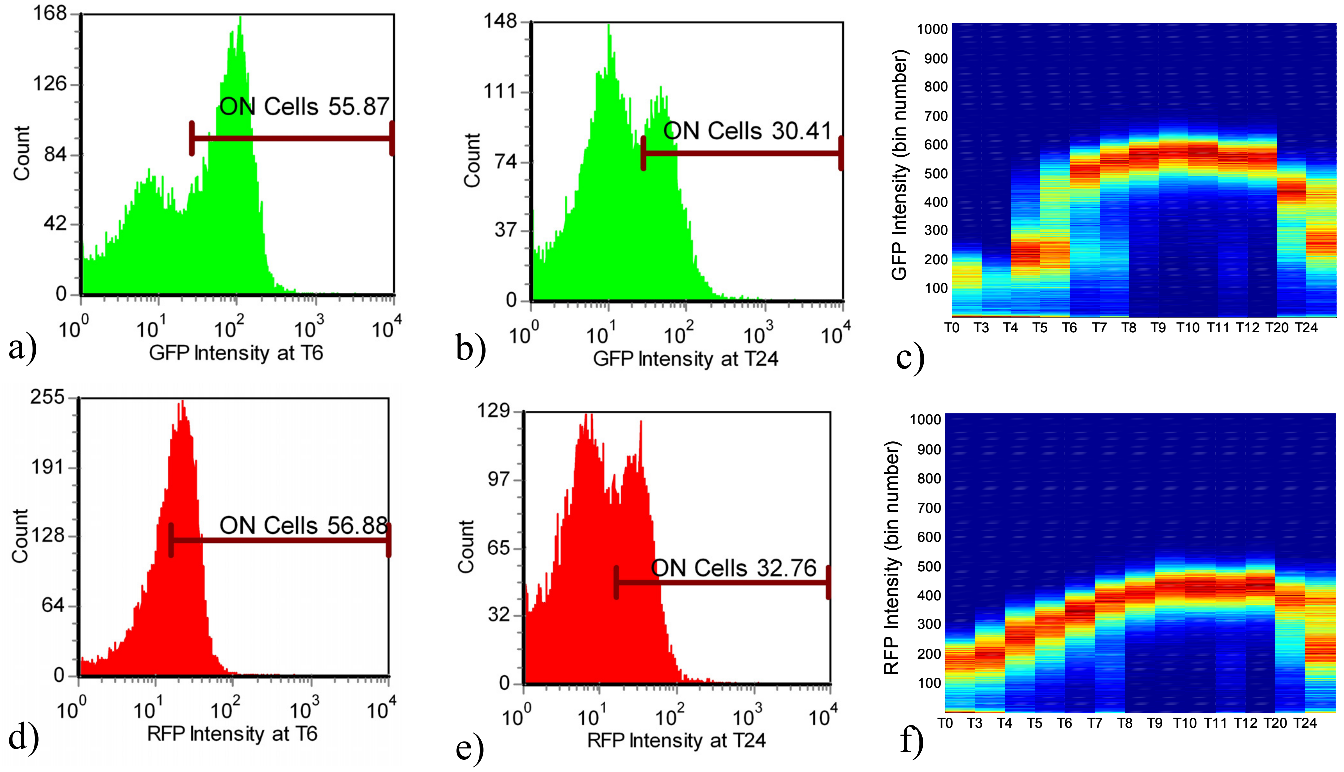

Figure S1. Histograms from flow cytometry analysis of *B. subtilis* cells with a history of growth in rich LB medium to a density of -2 (OD_{500}=1.016) prior to resuspension in starvation (SM) medium at time t0 at an initial density of OD_{600}=0.5. a) P*spoIIE-gfp* expression (GFP intensity) at 6 hours after t0; b) P*spoIIE-gfp* expression (GFP intensity) at 24hrs after t0; c) normalized heatmap of P*spoIIE-gfp* expression histograms (GFP intensity) sampled at selected time points from t0 to 24 hours after t0; d) P*aprE-dsred* expression (RFP intensity) at 6 hours after t0; e) P*aprE-dsred* expression (RFP intensity) at 24hrs after t0; f) normalized heatmap of P*aprE-dsred* expression (RFP intensity) sampled at selected time points from t0 to 24 hours after t0. In the heatmaps shown in (c,f), the distance along the y-axis is fluorescent intensity channel number (logarithmic scale), and the color along the y-axis corresponds to the (relative) number of cells (red corresponds to the maximum, yellow to an intermediate number of cells and dark blue to no cells).

Flow cytometry protocol:

Cells sampled at 10h and 20h after resuspension in SM medium were washed twice in one volume of 0.2 µM filtered PBS (phosphate-buffered saline, pH 7.4), 100x diluted in the same buffer and directly measured on a Partec CyFlow space flow cytometer (Partec GmbH, Münster Germany) operating an argon laser (488 nm). For each sample, at least 50,000 cells were analyzed. Data containing the green fluorescent signals were collected by a 520 nm BP filter

and the red fluorescent signals were collected by a 590 nm BP filter. Data was captured using Flomax 2.4f software (May 23 2006) and further analyzed using the commercial software package FCSExpress (http://www.denovosoftware.com). Background fluorescence was analyzed with parental strain *B. subtilis* 168 with each flow cytometric experiment to discriminate background from GFP and DsRed specific fluorescence. The heatmaps were constructed using the Matlab© functions surface.m, axis.m, and set.m.
