# Supplementary material for: Memory in Microbes: Quantifying History-Dependent Behavior in a Bacterium
Source: PLoS One. 2008 Feb 27;3(2):e1700. doi: 10.1371/journal.pone.0001700 (PMC2264733; doi:10.1371/journal.pone.0001700)
Supplement: Figure S2 — Sporulation initiation delay as a function of cell history. (0.13 MB PDF) [file pone.0001700.s008.pdf]

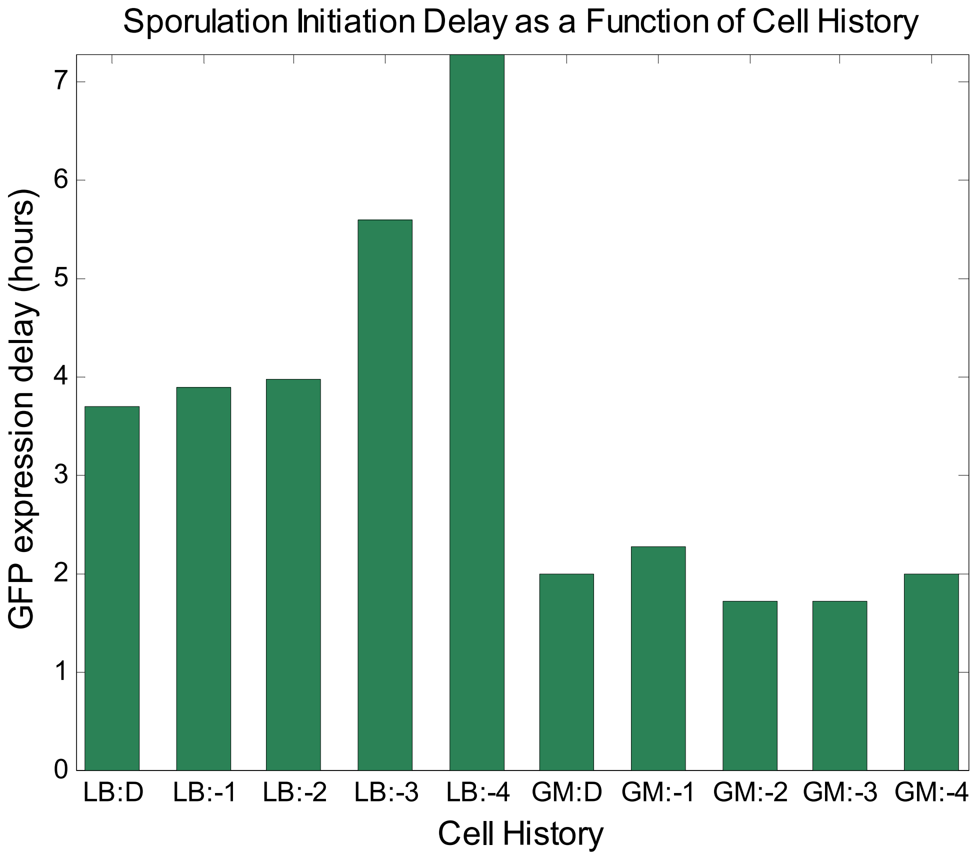

Figure S2. Sporulation initiation delay as a function of cell history. This plot shows sporulation initiation delay as a function of cell history as manifested in GFP expression delay in our *B. subtilis* reporter strain KEE. Cells were subjected to one of 10 cell histories prior to time t0 and then to a common stress condition, resuspension in SM, a sporulation 'starvation' medium, after t0 (see Table 1 for key to cell history labels). In theory at least, sporulation initiation *delay* is a likely feature of the sporulation regulation strategy of *B. subtilis* to exhibit memory, because of its potential to have a large impact on fitness. By calculating the mutual information between sporulation initiation delay times and total transient sporulation dynamics, we find that 85.95% of the transient memory exhibited by the sporulation initiation pathway is explained by cell-history modulation of sporulation initiation delay.
