# Supplementary material for: Memory in Microbes: Quantifying History-Dependent Behavior in a Bacterium
Source: PLoS One. 2008 Feb 27;3(2):e1700. doi: 10.1371/journal.pone.0001700 (PMC2264733; doi:10.1371/journal.pone.0001700)
Supplement: Figure S3 — The PspoIIE-gfp fusion activity tracks spoIIE gene expression. (0.25 MB PDF) [file pone.0001700.s009.pdf]

A.

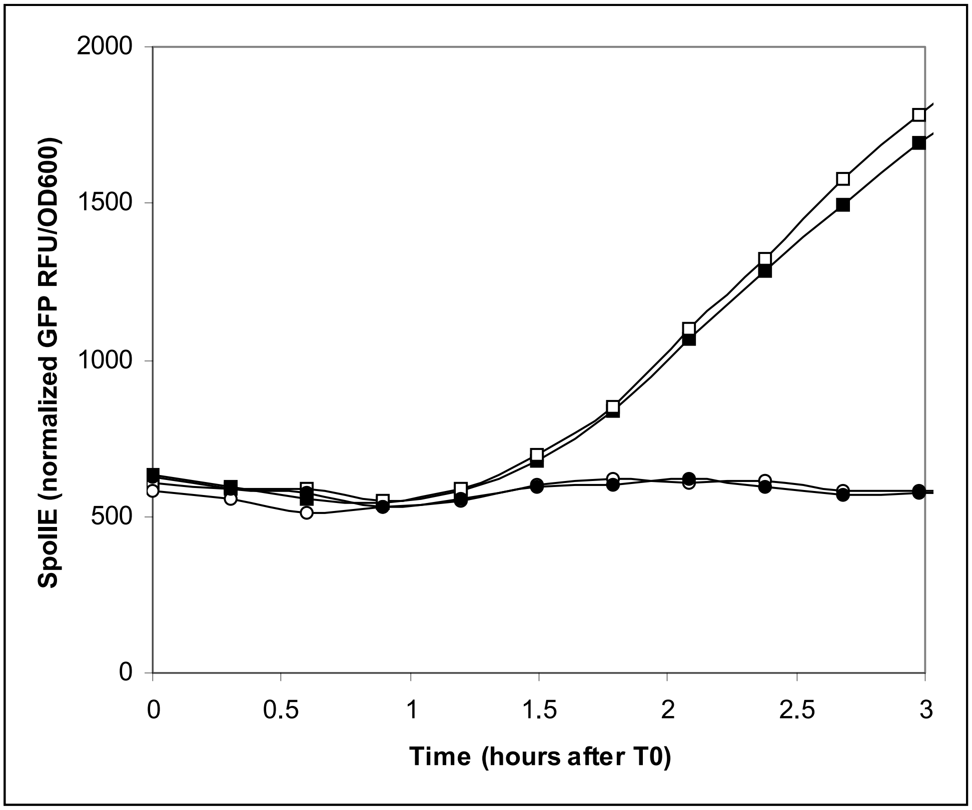

B.

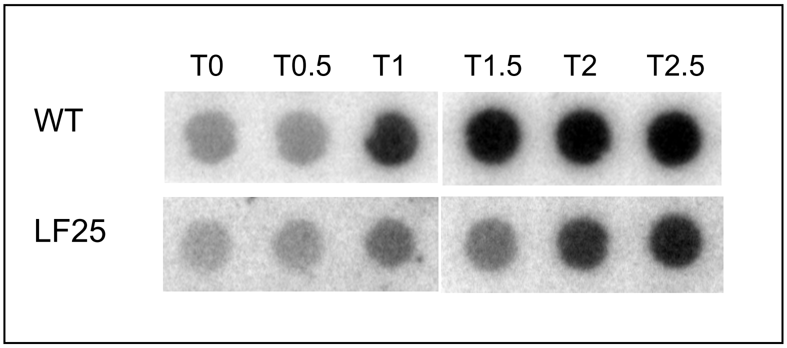

Figure S3. P*spoIIE-gfp* fusion activity compared to the *spoIIE* gene expression profile. This data shows that our GFP reporter for sporulation initiation in *B. subtilis* faithfully tracks expression of the stage II sporulation gene *spoIIE.* A. GFP activity in strain LF25 (*amyE::PspoIIE-gfp cmp*) and *spoIIE* transcriptional profile. Wild type and LF25 strains were grown in parallel in GM medium to an OD_{600} of 0.6 and 0.7 respectively. Cells were resuspended in SM medium to induce sporulation (as described in Materials and Methods) and two 200 µl aliquots were transferred into a microplate for time course measurement in a Safire spectrofluorimeter (TECAN inc.) with shaking, at 37ºC. Fluorescence (481 nm absorption and 507 nm emission) and OD_{600} were measured every 15 minutes. A. LF25 strain time points are shown with squares, whereas wild type *Bacillus subtilis* strain time points are shown with circles. Relative fluorescence (RFU) was normalized by the OD_{600}.

B. The transcriptional profile of the *spoIIE* gene was verified by total RNA dot-blot. Total RNA was extracted from *B. subtilis* cultures after induction of sporulation as previously described for *C. acetobutylicum*, [1]. RNA quality and quantity were checked by capillary electrophoresis using a 2100

Bioanalyzer (Agilent Technologies, Palo Alto, California). Total RNA samples (8µg each) taken just after resuspension (T0) and at 30 minutes intervals (T0.5, T1, T1.5, T2, T2.5) were spotted on positively charged Nylon membranes (Roche) using a dot blot manifold (Perkin Elmer). Denaturation, fixation on membrane and hybridization were performed as previously described for Northern blots [Fontaine, 2001 #15]. A radioactively labeled probe was PCR amplified within the *spoIIE* gene using primers spoIIE-D (cgtcggtaccATGGAAAAAGCAGAAAGAAGAG) and spoIIE-R (cctcggatccaccTGAAATTTCTTGTTTGTTTTGAA) on *B. subtilis* 168 genomic DNA as template. The resulting 689-bp fragment was radiolabeled as previously described [1].
The data shown in (B) confirmed *spoIIE* early and specific expression induction at the onset of sporulation, starting clearly from T1. The GFP activity of the P*spoIIE-gfp* fusion (squares) in (A) showed a clear increase starting from T1.5 after the resuspension event.

References

1. Fontaine L, Even S, Soucaille P, Lindley ND, Cocaign-Bousquet M (2001) Transcript quantification based on chemical labeling of RNA associated with fluorescent detection. Anal Biochem 298: 246-252
